# Psychotherapist remarks’ ML classifier: insights from LLM and topic modeling application

**DOI:** 10.3389/fpsyt.2025.1608163

**Published:** 2025-07-25

**Authors:** Alexander Vanin, Vadim Bolshev, Anastasia Panfilova

**Affiliations:** Laboratory of AI Technologies in Psychology, Institute of Psychology Russian Academy of Sciences, Moscow, Russia

**Keywords:** psychotherapy, therapist, language, speech, topic modeling, machine learning, BERTopic, ML classifier

## Abstract

**Introduction:**

This paper addresses the growing intersection of machine learning (ML) and psychotherapy by developing a classification model for analyzing topics in therapist remarks. Understanding recurring language patterns in therapist communication can enhance clinical practice, supervision, and training, yet systematic approaches to topic analysis remain limited.

**Methods:**

The study applies BERTopic, an ML-based topic modeling technique, to unstructured dialogues from two distinct groups of therapists: classical (founders of therapeutic schools such as Carl Rogers, Fritz Perls, and Albert Ellis) and modern practitioners representing diverse psychotherapeutic approaches. The implementation involves constructing a vector space of therapist remarks, applying dimensionality reduction, clustering, and optimizing topic representations. To improve interpretability, expert assessment and manual refinement complement the automated modeling process. The resulting topics are used as features to train an ML classifier, which is then tested on a case study comparing Carl Rogers’ sessions with those of modern Cognitive Behavioral Therapy (CBT) practitioners.

**Results:**

The analysis identifies the most common and stable topics across both therapist groups, highlighting recurring patterns and unique thematic compositions. The case study reveals distinct differences in thematic structures, with key topics emerging that characterize each group’s therapeutic discourse. The trained classifier demonstrates robust performance in distinguishing these thematic patterns.

**Discussion:**

The study shows that automated topic modeling, combined with expert input, can effectively uncover how therapist language patterns emerge and persist across different therapeutic styles. The resulting model, made publicly available, offers broad applications in psychotherapy research, clinical supervision, and training. These findings underscore the potential of topic modeling as a valuable tool for deepening our understanding of therapist communication and advancing ML applications in psychotherapy.

## 1 Introduction

Language plays a central role in psychotherapy, shaping the therapeutic process and influencing client outcomes. The way therapists communicate – through their interventions, strategies, and speech patterns – provides valuable insights into therapy’s effectiveness. Traditionally, analyzing therapist communication required manual coding, an intensive process susceptible to bias and scalability limitations (1). However, the advent of machine learning (ML) tools offers a transformative approach to this challenge. By automating the analysis of vast amounts of data, ML algorithms can identify subtle patterns and nuances in therapeutic communication that would be impossible to detect manually. This opens up new avenues for understanding and optimizing therapeutic techniques.

Building upon the potential, the application of ML tools has already yielded significant advancements in psychological counseling and psychotherapy, demonstrating its practical utility in diverse clinical contexts. For example, Alexopoulos et al. (2) used ML to identify predictors of suicidal ideation trajectories in older adults undergoing brief psychotherapy. Employing LASSO regression, random forest, gradient boosting, and classification tree models, they found that hopelessness, neuroticism, and low self-efficacy were the strongest predictors of an unfavorable trajectory. Similarly, Wallert et al. (3) applied supervised ML with multi-modal data to predict remission of major depressive disorder following Internet-based Cognitive Behavioral Therapy (CBT), incorporating demographic, clinical, process-related, and genetic predictors.

A particularly promising application of ML in psychotherapy is the analysis of therapist-client dialogue, including topic modeling – an unsupervised natural language processing (NLP) method that extracts abstract topics from text corpora. Topic modeling can identify commonly used therapeutic techniques (e.g., empathy, reframing, cognitive restructuring), supporting clinical practice, supervision, and training (4). It also enables tracking shifts in therapeutic approaches over time, facilitating data-driven assessments of treatment progress and adaptations to clients’ evolving needs (5).

Several studies have investigated the use of topic modeling on psychotherapy session analysis, leveraging ML algorithms. Gaut et al. (6) automated psychotherapy session coding using Labeled Latent Dirichlet Allocation (L-LDA) and LASSO regression, demonstrating that L-LDA outperformed LASSO in predicting symptom-related talk-turns at the session level. Atzil-Slonim et al. (7) applied NLP to analyze psychotherapy sessions, using Latent Dirichlet Allocation (LDA) topic modeling to examine key themes in session transcripts and their relationship with client outcomes. Lin et al. (8) further advanced this approach by comparing neural topic modeling methods to analyze psychiatric conditions in psychotherapy, incorporating temporal modeling to track topic evolution at the turn level. Their findings revealed that patient and therapist session trajectories were more distinct in anxiety and depression sessions but more intertwined in schizophrenia sessions. Topic modeling can also be dynamic, as demonstrated by the LDASeq-based approach proposed by Levis et al. (9), which distinguishes patients who died by suicide by identifying changes in engagement, expressivity, and therapeutic alliance as key differentiators. Unlike traditional static methods, this approach takes into account temporal dynamics, allowing for tracking how topics emerge, evolve, and dissipate over time, which can reveal important patterns in the psychotherapy.

Of particular interest are transformer-based topic modeling approaches. Thus, Gao and Sazara (10) leveraged BERTopic – a transformer-based topic modeling technique (11) – to analyze mental health research abstracts and demonstrated its superior topic coherence and diversity compared to traditional methods like Top2Vec and LDA-BERT. Additionally, Ji et al. (12) introduced domain-specific pretrained language models (MentalBERT, MentalRoBERTa), significantly improving performance in mental disorder detection tasks.

Milligan et al. (13) applied the transformer-based model in digital mental health interventions and predictive modeling. The authors combined the RoBERTa large language model in conjunction with topic modeling as well as clinical expertise to develop a granular outcome measure (Adult SWAN-OM) for single-session therapy, ensuring relevance and clarity for both clinicians and service users. Lalk et al. (14) applied BERTopic to psychotherapy transcripts to predict symptom severity and therapeutic alliance, identifying key topics using explainable AI (XAI). Analyzing 552 transcripts from 124 patients, they extracted 250 topics from both patient and therapist speech. Their findings indicated that patient speech better predicted symptom severity, whereas therapist speech was more predictive of alliance. Gunal et al. (15) explored the potential of large language models (LLMs) in mental health care, employing a decision transformer architecture to recommend topics during therapy sessions via offline reinforcement learning.

Our research builds on these advancements by focusing specifically on therapist remarks in psychotherapy. While many studies analyze patient language or general therapist-client interactions, our study shifts the emphasis to therapists’ speech, systematically identifying and interpreting therapeutic communication strategies. Unlike previous studies that extracted broad conversational topics [e.g., music, planting, eating, washing as defined in (14)], our work directly links therapist remarks to therapeutic strategies, providing clinically actionable insights. Using BERTopic, we analyze therapist remarks to uncover the intent behind his/her statements, offering a novel perspective on communication strategies and their role in therapy. Furthermore, we develop an ML model to automate topic detection, contributing to the growing body of research on ML applications in psychotherapy and enhancing our understanding of how therapists’ verbal techniques influence the therapeutic process.

## 2 Materials and methods

### 2.1 Datasets

The primary source material consisted of publicly available recordings of psychotherapeutic sessions posted on YouTube. In addition, textual transcripts of Carl Rogers’ sessions (16, 17) were used. Full information on the materials used, including source links, is listed in **Supplementary Table A.1** of **Appendix A**. The materials were divided into two datasets: sessions conducted by classical and by modern therapists. The sample of classical psychotherapeutic sessions includes 25 transcripts and recordings, while the sample of modern sessions comprises 97 ones. Classical therapists are represented by session transcripts and recordings of the founders of therapeutic schools – Carl Rogers, Fritz Perls, and Albert Ellis – while modern therapists are represented by recordings of 37 unique practitioners. The samples of classical and modern psychotherapeutic sessions involved 22 and 66 unique clients, respectively. Sessions conducted by modern therapists represent a range of psychotherapeutic approaches, with Cognitive Behavioral Therapy (CBT) being the most prevalent, accounting for 27 sessions. After conducting speaker diarization and transcription of the recordings, 8641 remarks were obtained for classical therapists and 4058 for modern ones, all of which are publicly available at DataPoint (18).

### 2.2 Study design

The acquired document corpora were subjected to text preprocessing prior to the investigation, which included segmentation of remarks into separate sentences, performing lexical normalization, cleaning metadata, and converting to a unified case.

Subsequently, topic modeling was applied to the preprocessed corpora using the BERTopic, a ML-based topic modeling tool. Pre-trained embeddings from the Sentence-Transformer model ‘paraphrase-multilingual-MiniLM-L12-v2’ were used to create a vector space. UMAP (Uniform Manifold Approximation and Projection) method was applied to reduce the vector space’s dimensionality, and HDBSCAN (Hierarchical Density-Based Spatial Clustering of Applications with Noise) was used to cluster the data. The topic representation of the clusters was made using BERTopic’s built-in c-TF-IDF method for assessing the importance of words within the context of document clusters, and its optimization was primarily achieved through the use of large language models like GPT.

After the topic modeling process, the results underwent expert analysis, followed by the removal or merging of topics so as to achieve a more interpretable topic structure of therapist remarks. Subsequently, a detailed interpretation of topic clusters was conducted for each corpus of remarks from classical and modern therapists. The most semantically similar topics between the two corpora were merged to create one topic model. In the final phase of the research, a ML classification model for analyzing therapist remarks within a psychotherapeutic context was trained on the merged dataset and then tested on a case study.

### 2.3 Topic modeling with BERTopic

Two text data corpora were under topic modeling in parallel using the BERTopic algorithm, which is a multi-stage topic modeling process. The initial stage is the construction of a vector space for each document. A dimensionality reduction technique is used to the resultant vector space in order to increase the effectiveness of further computations and visualization. After that, documents are grouped by topic proximity using a clustering technique. At the final stage, text descriptions of the resulting topics are formed and optimized. A more detailed description of each stage is given below.

#### 2.3.1 Data corpus preparation

To prepare the corpora for topic modeling, a set of preprocessing operations was carried out using the spaCy library models pretrained on the OntoNotes Release 5.0 corpus (19) with the additional resources of ClearNLP Constituent-to-Dependency Conversion (20) and WordNet (21). The following stages were performed during corpora processing:

- segmentation of remarks consisting of splitting the continuous text of remarks into separate sentences in order to isolate syntactic units;
- lexical normalization, which included decoding abbreviations (replacing abbreviated forms of modal verbs and negative particles with their full forms to ensure homogeneity of the vocabulary), unification (removal of uninformative elements, such as interjections, speech fillers and other stop words) and bringing some lexical units to a single orthographic form (for example, «okay»);
- cleaning from metadata, which included removing time pause marks (excluding information from the text that indicates the duration of pauses), as well as eliminating identifiers (removing question numbers and other identifiers that do not carry semantic load) in order to preserve only linguistic information;
- bringing to a unified register consisting of converting all letters to lower case so as to unify the text and eliminate the influence of the register on further analysis.

#### 2.3.2 Vector space construction

We created the vector space from document embeddings obtained using language neural networks with the transformer architecture, hence allowing not only to effectively take into account semantic relationships in texts, but also to avoid many stages of text preprocessing, such as removing stop words, stemming or lemmatization (22, 23). We applied pre-trained multilingual embeddings of the Sentence-Transformer model ‘paraphrase-multilingual-MiniLM-L12-v2’ so as to obtain embeddings of the considered text corpora. The choice of this model was due to its higher efficiency compared to others, which was verified empirically, including by comparing with specialized models, for example, BIOBERT, trained on medical texts.

#### 2.3.3 Vector space dimensionality reduction

Since the vector space of embeddings is usually a sparse matrix, BERTopic provides the option to apply a variety of dimensionality reduction algorithms, such as PCA, t-SNE or UMAP (used by default). According to studies (24), this step allows expediting the clustering process and increasing its accuracy. Based on the results of (25), in our study, we chose the UMAP (Uniform Manifold Approximation and Projection) method. Metric used to calculate the distances between points in the original space was Cosine Proximity.

#### 2.3.4 Vector space clustering

A wide range of clustering algorithms are supported by BERTopic, including contemporary density-based techniques like HDBSCAN and more conventional techniques like k-means and agglomerative clustering. HDBSCAN was used for the current research because it offered the most reliable and accurate text data clustering This algorithm can efficiently handle variable-density data and automatically detect outliers (24). We used the Euclidean distance to calculate the proximity between documents in this technique and assumed that a cluster had a minimum of 40 objects.

#### 2.3.5 Topic representation of clusters

A distinctive feature of the BERTopic algorithm from other topic modeling algorithms (e.g., Top2Vec) is its ability to form semantically rich representations of topics. To do this, a single bag-of-words vector is constructed for each cluster and then a list of the most significant words in the cluster is derived using the c-TF-IDF metric (26). This metric is an extension of traditional TF-IDF adapted for cluster analysis and allows assessing the importance of terms (words) in the context of a specific cluster.

Determining the importance of terms makes it possible to create a list of the most relevant keywords describing a certain cluster, which is the topic representation of the cluster in question. This opens up options for optimizing the cluster structure by combining topics with similar semantic content, hence reducing the number of clusters and minimize the number of outliers. In addition to a relative accurate representation of clusters, BERTopic enables the optimization of topic representation by using built-in additional techniques. These include methods based on keywords (KeyBERTInspired), maximum marginal relevance (MMR), part-of-speech analysis (using the spaCy library), and the application of large language models (e.g., GPT or T5). The latter are particularly promising due to their ability to automate the process of generating cluster descriptions and significantly lessen the workload of experts.

We combined all of the aforementioned techniques in our study so as to further personalize the topic representations. In order to generate a description of each cluster, we used the following prompt to the «GPT-4o mini» model:

“I have a topic that contains the following documents:

[DOCUMENTS]

The topic is described by the following keywords: [KEYWORDS]

Based on the information above, extract a short but highly descriptive topic label of at most 5 words. Make sure it is in the following format:

topic: <topic label>

Topic must be in the language in which the documents are written”

#### 2.3.6 Assessment and optimization of topic modeling outcomes

Following the topic modeling procedure, the resulting topics were subjected to expert evaluation to enhance the interpretability of the thematic structure of therapist remarks. The expert panel included a psychologist with a PhD and an original degree in clinical psychology, as well as a PhD in technical sciences specializing in psychology. Decisions regarding the retention or exclusion of specific topics were based on their relevance to the therapeutic process – such as their alignment with clinically meaningful interventions or therapist intentions – and their interpretability within the context of psychotherapeutic discourse. Topic merging was guided by the proximity of topics in hierarchical cluster analysis and further supported by qualitative content analysis of representative topic texts to identify the presence of a shared overarching topic. In addition to visual assessment, topic structure was further evaluated using the c_v (Coherence Value) metric, as proposed in (27), to quantify topic diversity. This metric assesses the semantic relatedness of the top words within a given topic, with values ranging from 0 to 1, where a score closer to 1 indicates stronger semantic coherence. Following the thematic structure evaluation, decisions were made regarding topic removal or merging to achieve a more interpretable structure for therapist remarks. Three iterative cycles of actions described above were required to attain optimal topic structures for the datasets analyzed. Subsequently, the experts provided a detailed interpretation of each topic cluster and the most semantically similar topics between the two corpora identified by calculating cosine similarity.

### 2.4 Development of topic ML classifier

Subsequent research focused on developing a ML classifier for therapist remarks within psychotherapeutic contexts, irrespective of the therapist’s orientation (classical or modern). This required a combined dataset labeled using a merged BERTopic model. This merged model was created from separate BERTopic models trained on remarks’ corpora from classical and modern therapists. The merging process involved comparing the cosine similarity between topic embeddings from the two models. So as to merge topics, a minimum cosine similarity threshold was chosen equal to 0.7. This threshold was selected as optimal based on the results of third-party studies (28, 29) and our own empirical experience, as it produced a coherent and meaningful general thematic structure. Topics from the classical therapist corpus served as the primary set. Each topic from the modern therapist corpus was compared against primary topics. If the cosine similarity exceeded 0.7, the two topics were merged, while the unification always took place with the topic exhibiting the highest similarity score. Conversely, if no such similarity was found above the threshold, the modern therapist topic was added as a distinct entity to the primary set.

A labeled dataset, derived from the merged topic structure, was prepared for the multiclass classification task of identifying topics within individual remarks. This dataset was subsequently partitioned into training, validation, and testing subsets with a 70%-15%-15% ratio, stratified by category. Given the prior application of BERT embeddings for topic clustering, it was hypothesized that fine-tuning the BERT model could yield sufficient accuracy for the subsequent topic classification task. Consequently, the bert-base-uncased model from Google (30), comprising 110 million parameters, was selected as the foundational model. Classification performance was evaluated using the F1-measure metric, computed for each subset, to assess the efficacy of the ML classifier on individual topics.

## 3 Results

### 3.1 Topic structures of classical and modern psychotherapeutic sessions

#### 3.1.1 Optimization of topic structures

Applying the BERTopic topic modeling algorithm to two text corpora – containing remarks by classical and modern therapists – initially resulted in 95 and 110 topic clusters, respectively. For the obtained topic structures, the topic diversity was 0.429 for classical therapists and 0.377 for modern therapists, both of which are considered good results for real-world topic modeling data (27).

Through expert interpretation of the topic modeling results, we were able to identify a large number of similar clusters in terms of the subjects’ meanings. These clusters were either classified as close in cosine distance by the BERTopic model or distant, but still having similar semantic content by the expert view. In both cases, these topics displayed a sufficient number of representative documents to be considered separate clusters (as indicated above, the minimum number of documents in a cluster was taken to be 40). So as to combine the clusters we applied the merge_topics method (built into the model) creating a new cluster by averaging the vectors of the merged topics. In addition, the clusters consisting of filler words and discourse markers with little semantic weight (such «yes», «Uhm-hm», «Is that…?», etc.) were also merged into a distinct category called «Others».

Following manual refinement of the thematic structures based on expert knowledge, 43 and 46 clusters were identified in the remarks of classical and modern therapists, respectively. At the same time, the coherence of topics increased, as expected, to 0.470 (classical therapists) and 0.431 (modern therapists).

#### 3.1.2 Interpretation of topic structures

In the research we examined the identified topics in the therapists’ remarks sequentially, referencing their representation in the results of hierarchical cluster analysis (**Supplementary Figures B1**, **B2**, **Appendix B**) which determines the proximity of topics to each other. Due to space limitations in the article, we thoroughly discussed the topics associated with classical therapists, followed by an exploration of the topics associated with modern therapists in **Appendix B** (**Supplementary Tables B1**, **B2**, respectively), where the main characteristics of the topics are presented, including the topic description and examples of the topic in the therapist’s speech.

**Figure 1:**
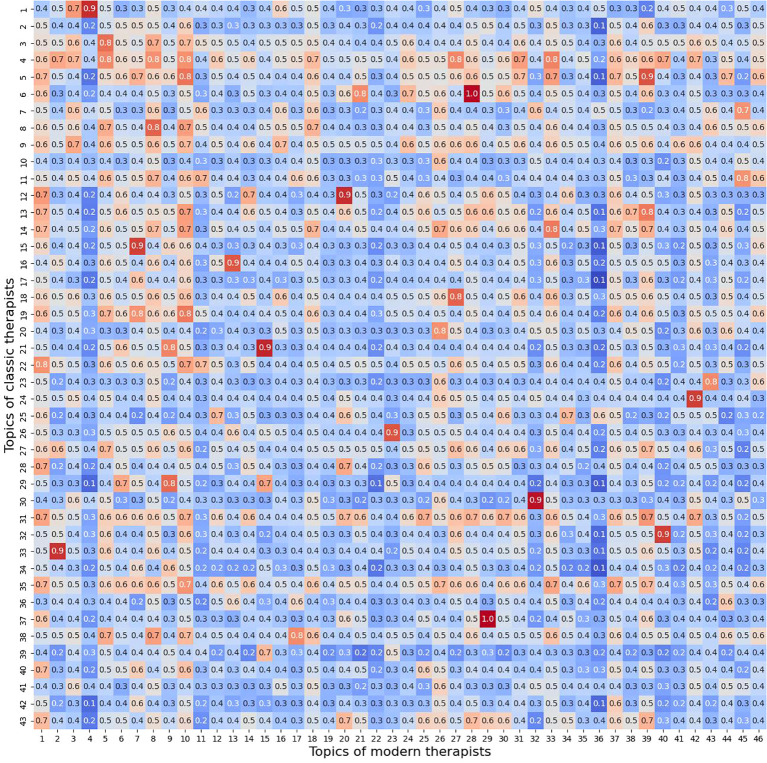
Heatmap of cosine similarity between topics of classical and modern therapists.

We also identified the most similar topics in the discourse of classical and modern therapists, reflecting the main problems of clients. The proximity of topics between both groups of therapists was determined based on cosine similarity (**Figure 1**). This method calculates the cosine of the angle between two vectors in a multi-dimensional space, measuring how similar the topics are in terms of their content and context. By applying this technique, we could quantify the degree of similarity between the topic representations of classical and modern therapists, allowing for a comparative analysis of their speech patterns.

Based on the calculated cosine similarity, we could conclude that prominent topics in the discourse of both therapist groups, likely reflecting core client concerns, include emotional experiences, familial and peer relationships, as well as issues related to personal development, self-esteem, and other psychological challenges.

### 3.2 Machine learning classifier on merged topic structure

To develop a machine learning (ML) classifier for therapist remarks, it was necessary to integrate two distinct topic structures. This integration of topic models was achieved by assessing the cosine similarity between individual topics. The merging process resulted in 37 coherent topics (see **Appendix C**, **Supplementary Table C1** for details on the merging), which were subsequently organized into 8 overarching topic groups for simplified analysis. The detailed composition of these groups is presented in **Table 1**. Comprehensive descriptions of the identified topics, along with illustrative examples from therapist remarks, are available in **Appendix C**, **Supplementary Table C2**. Therapeutic strategies and techniques derived from the analysis of the merged topic structure are discussed in the dedicated Discussion section.

**Table 1:** Topic groups and the included topics.

Topic group	Description of topic group	Included topics
I. Therapy Process and Communication (6 topics).	Covering the structure of therapy sessions, therapist-client communication, and therapeutic goals.	1. Time Up and Future Meetings. 7. See and Understanding Conversations. 8. Clarifying Meaning and Intent. 11. Open Conversation and Sharing. 19. Voices and Perception of Sound. 31. Therapy and Father Relationships.
II. Emotions, Fears, and Emotional Regulation (8 topics).	Exploring the client's emotional experiences, including fear, anger, sadness, joy, and stress, as well as coping mechanisms.	2. Complex Emotions Toward Him. 6. Understanding and Confronting Fear. 20. Difficulties and Emotional Burdens. 21. Fear and Reflection on Aging. 22. Emotions of Crying and Tears. 29. Expressions of Anger and Frustration. 32. Expressions of Happiness and Joy. 37. Nervous System and Stress Response.
III. Self-Perception, Identity, and Inner Conflict (6 topics).	Addressing self-esteem, self-acceptance, identity, internal conflicts, and feelings of guilt.	5. Self-Acceptance and Relationships. 13. Guilt and Self-Blame Dynamics. 16. Gender Roles and Relationships. 17. Struggles with Personal Change. 25. Inner Struggle and Helplessness. 30. Nurturing the Inner Child.
IV. Relationships and Social Dynamics (4 topics).	Focusing on the client's relationships with family, friends, partners, and colleagues, as well as broader social interactions.	14. Dynamics of Meaningful Relationships. 18. Complex Mother-Sibling Relationships. 23. Father-Child Relationships and Authority. 28. Marriage Anxiety and Dependence.
V. Personal Growth, Life Direction, and Decision-Making (6 topics).	Related to personal development, finding life direction, decision-making processes, and exploring new opportunities.	3. Desires and Disappointments. 4. Personal Growth and Decision-Making. 9. Desire to Escape and Leave. 10. Uncertainty and Understanding Issues. 24. Possibilities and Potential Outcomes. 26. Pursuing Meaningful Personal Goals.
VI. Work, Education, and Career (2 topics).	Covering aspects of professional life, educational experiences, and career aspirations.	15. Struggles and Desires in Learning. 27. Job Anxiety and Self-Reflection.
VII. Past Experiences and Their Influence (2 topics).	Analyzing how past experiences shape the client's current psychological state.	12. Exploring Emotional Hurt and Bitterness. 33. Revisiting the Past Together.
VIII. Health and Well-Being (3 topics).	Addressing aspects of physical and mental health, including depression, alcohol use, and energy management.	34. Drinking Habits and Concerns. 35. Managing and Increasing Energy Levels. 36. Understanding Depression and Its Roots.

Training an ML model capable of identifying the remark topics described above was carried out by fine-tuning Google’s BERT-base-uncased model (30), which has 110M parameters. The model was trained with the following hyperparameters: a batch size of 32, a learning rate of 2e-5, and a total of 10 epochs. The value of the learning rate parameter of 2e-5 demonstrates the greatest stability in BERT fine-tuning tasks, according to (26). During the training process, overfitting of the model was observed at the end of the procedure over 10 epochs, thus, the model demonstrating the maximum accuracy values ​​on the validation sample was selected as the best one. The overall F1-measure metric with macroaveraging and weighted average for each subset (train, validation, test) is presented in **Table 2**.

**Table 2:** Score metrics for trained classifier.

Subset	Macro F1	Weighted F1
Test	0.74	0.75
Validation	0.77	0.77
Train	0.97	0.98

## 4 Discussion

This section addresses three aspects pertaining to the machine learning (ML) classification of therapist remarks by topic. First, it provides a description of therapeutic strategies and techniques derived from the established topic structure, which served as labels for the ML classifier. Second, it presents an assessment of the ML model’s classification performance on a per-topic basis. Finally, it concludes with a focused case study illustrating the classifier’s application – specifically, a comparison of the topic structure in Carl Rogers’ sessions and those from Cognitive Behavioral Therapy (CBT) sessions conducted by modern therapists.

### 4.1 Analysis of topic structure

The analysis of the therapist’s speech within the merged topic structure provides insights into applied therapeutic strategies and techniques. One such strategy is supporting the dynamics of the therapeutic process, where the therapist demonstrates readiness to address the client’s problematic situation (7 «See and Understanding Conversations»). The therapist also strives to build trust in the conversation with the client (11 «Open Conversation and Sharing»).

Another commonly observed technique is normalization, such as acknowledging the client’s fear (6 «Understanding and Confronting Fear») or validating client’s emotions regarding the complexity of the situation (20 «Difficulties and Emotional Burdens»). In doing so, the therapist not only applies various techniques for working with fear but also motivates the client to confront challenging circumstances.

The therapist also demonstrates empathy and support (22 «Emotions of Crying and Tears») and legitimizes the client’s desires – for example, the need to feel anger – and discusses ways to manage it (29 «Expressions of Anger and Frustration»). Additionally, the therapist suggests methods for coping with stress (37 «Nervous System and Stress Response»), potential strategies for overcoming helplessness (25 «Inner Struggle and Helplessness»), working through emotional pain (12 «Exploring Emotional Hurt and Bitterness»), and various approaches to addressing depression (36 «Understanding Depression and Its Roots»). The therapist also employs reframing techniques to reduce the client’s anxiety (27 «Job Anxiety and Self-Reflection»). Furthermore, the therapist actively encourages the client by highlighting his/her positive attitude and progress in therapy (32 «Expressions of Happiness and Joy») and emphasizing internal motivation (15 «Struggles and Desires in Learning»).

A significant portion of the therapist’s speech is dedicated to analyzing and interpreting the underlying causes of various emotional states and behaviors, such as anxiety (6 «Understanding and Confronting Fear»), anger responses (29 «Expressions of Anger and Frustration»), sources of self-blame (13 «Guilt and Self-Blame Dynamics»), emotional ambivalence (2 «Complex Emotions Toward Him»), and addiction-related concerns (34 «Drinking Habits and Concerns»). The therapist also examines procrastination (17 «Struggles with Personal Change»), indecision in life choices (4 «Personal Growth and Decision-Making»), and various aspects of the client’s relationships with others (14 «Dynamics of Meaningful Relationships»), including dependence on external opinions (5 «Self-Acceptance and Relationships»), challenges related to gender roles (16 «Gender Roles and Relationships»), and hidden motives for entering marriage (28 «Marriage Anxiety and Dependence»). These interpretations are often accompanied by the use of metaphors (19 «Voices and Perception of Sound», 30 «Nurturing the Inner Child»).

Overall, each topic group reflects a combination of strategies – such as empathy, normalization, interpretation, metaphor use, and structuring – that enable the therapist to adapt to the client’s needs and strengthen the therapeutic alliance.

### 4.2 Per-topic analysis of ML classifier performance

**Figure 2:**
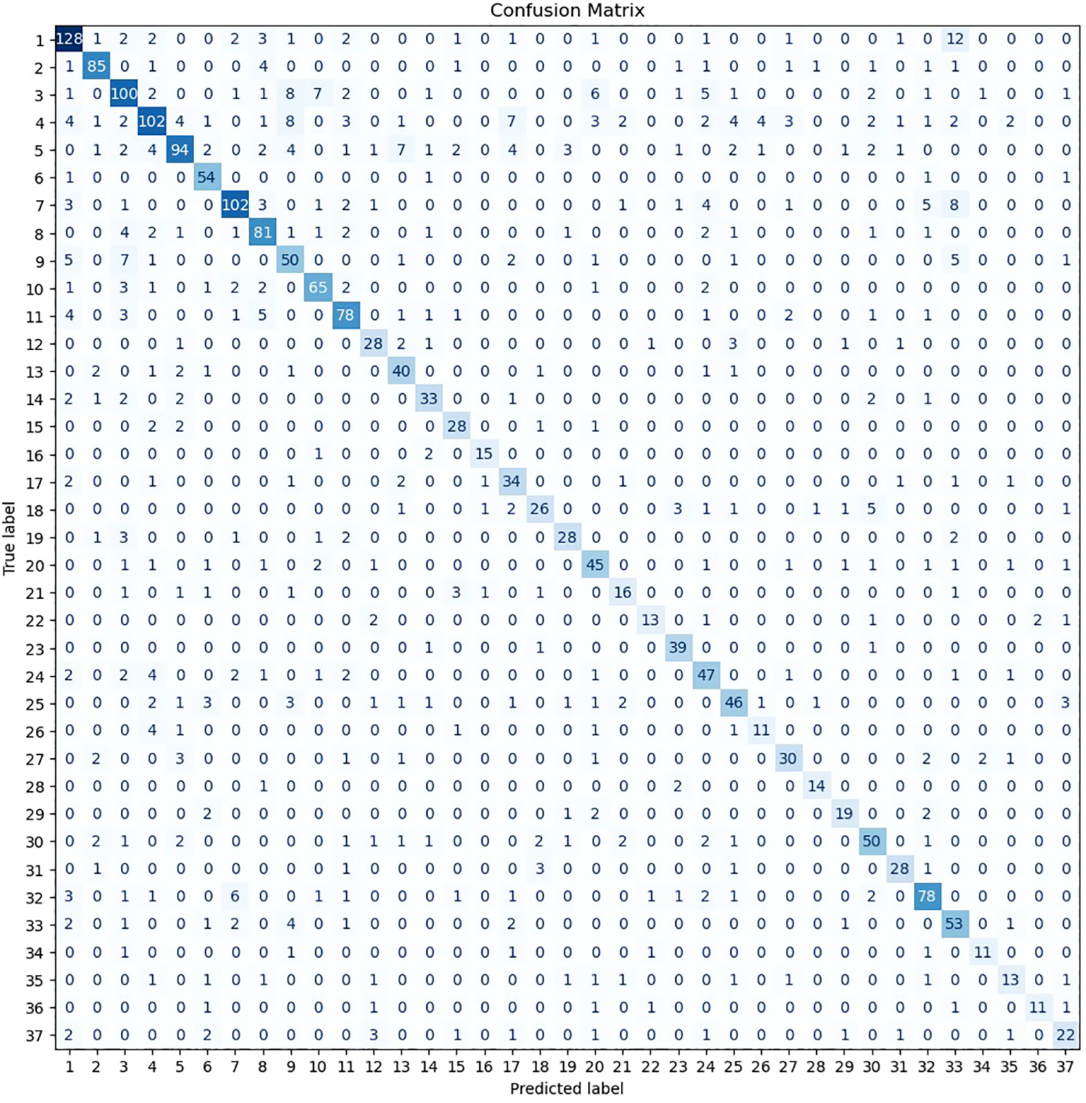
Confusion matrix for therapist remark topics’ classification on the test subset.

To assess the ML model’s ability to classify therapist remark topics, the F1-measure was computed for each relevant topic on the test subset. **Table 3** summarizes these performance metrics while **Figure 2** provides a visual representation of the model’s classifications through a confusion matrix diagram.

**Table 3:** Score metrics for test subset by categories.

ID	Topics	F1-mesure
1	Time Up and Future Meetings	0.80
2	Complex Emotions Toward Him	0.87
3	Desires and Disappointments	0.72
4	Personal Growth and Decision-Making	0.70
5	Self-Acceptance and Relationships	0.75
6	Understanding and Confronting Fear	0.84
7	See and Understanding Conversations	0.81
8	Clarifying Meaning and Intent	0.79
9	Desire to Escape and Leave	0.64
10	Uncertainty and Understanding Issues	0.81
11	Open Conversation and Sharing	0.78
12	Exploring Emotional Hurt and Bitterness	0.72
13	Guilt and Self-Blame Dynamics	0.74
14	Dynamics of Meaningful Relationships	0.75
15	Struggles and Desires in Learning	0.77
16	Gender Roles and Relationships	0.83
17	Struggles with Personal Change	0.67
18	Complex Mother-Sibling Relationships	0.67
19	Voices and Perception of Sound	0.76
20	Difficulties and Emotional Burdens	0.71
21	Fear and Reflection on Aging	0.63
22	Emotions of Crying and Tears	0.70
23	Father-Child Relationships and Authority	0.86
24	Possibilities and Potential Outcomes	0.68
25	Inner Struggle and Helplessness	0.69
26	Pursuing Meaningful Personal Goals	0.61
27	Job Anxiety and Self-Reflection	0.71
28	Marriage Anxiety and Dependence	0.82
29	Expressions of Anger and Frustration	0.75
30	Nurturing the inner child	0.72
31	Therapy and Father Relationships	0.81
32	Expressions of Happiness and Joy	0.78
33	Revisiting the Past Together	0.68
34	Drinking Habits and Concerns	0.73
35	Managing and Increasing Energy Levels	0.58
36	Understanding Depression and Its Roots	0.73
37	Nervous System and Stress Response	0.64

The model demonstrated the highest classification accuracy for topics 2 «Complex Emotions Toward Him» and 23 «Father-Child Relationships and Authority». Topic 2 is based on themes from classical therapists, focusing on discussions related to emotional experiences in relationships with men. Topic 23 integrates themes from both classical and modern therapists and encompasses issues such as family dynamics, identity, and relationships with fathers and stepfathers. Another notable category is topic 6 «Understanding and Confronting Fear», which also draws from both classical and modern therapeutic traditions. This topic includes statements addressing the understanding and normalization of fear, its interpretation, coping strategies, and its impact on mental health. Topic 16 «Gender Roles and Relationships» originates from classical therapist themes and involves discussions of masculinity, femininity, and their roles in interpersonal relationships.

In contrast, the topic with the lowest classification accuracy was Topic 35 «Managing and Increasing Energy Levels», which is based on themes from modern therapists and includes discussions on energy levels for daily activity and methods for increasing life energy.

The model trained in this study is publicly available (31), making it possible to apply it practically in similar research. It’s worth noting that the model’s accuracy in identifying categories doesn’t depend on whether they were formed from classical therapists only, modern therapists only, or a combination of both. However, separating sessions into classical and modern therapists allowed us to identify unique topics that might have been considered outliers by the algorithm if the sessions hadn’t been separated.

### 4.3 Case study of classifier application

Although the developed model lends itself to various applications, due to limitations of our dataset, we chose to conduct a topic analysis comparing the compositional structure of remarks in Carl Rogers’ sessions with those from Cognitive Behavioral Therapy (CBT) sessions conducted by modern therapists. The selection of Carl Rogers was motivated by two primary factors. First, the classical therapist sample is predominantly composed of Rogers’ remarks. Second, Rogers’ pivotal role as a founder and leading figure in humanistic psychology – particularly in the development of client-centered therapy – suggests that the topic profile of his remarks may exhibit features distinctive to this psychotherapeutic tradition. Meanwhile, the selection of the CBT approach was driven by its prevalence in the sample of sessions conducted by modern therapists. To mitigate the disparity in sample sizes between Rogers’ and CBT remarks, a rank-based approach was implemented. Specifically, the percentage of remarks corresponding to each topic within both Rogers’ and CBT samples was determined and subsequently ranked. In cases of identical percentage values, average ranks were assigned, with the topic exhibiting the highest percentage of inclusion receiving a rank of 1.

**Figure 3:**
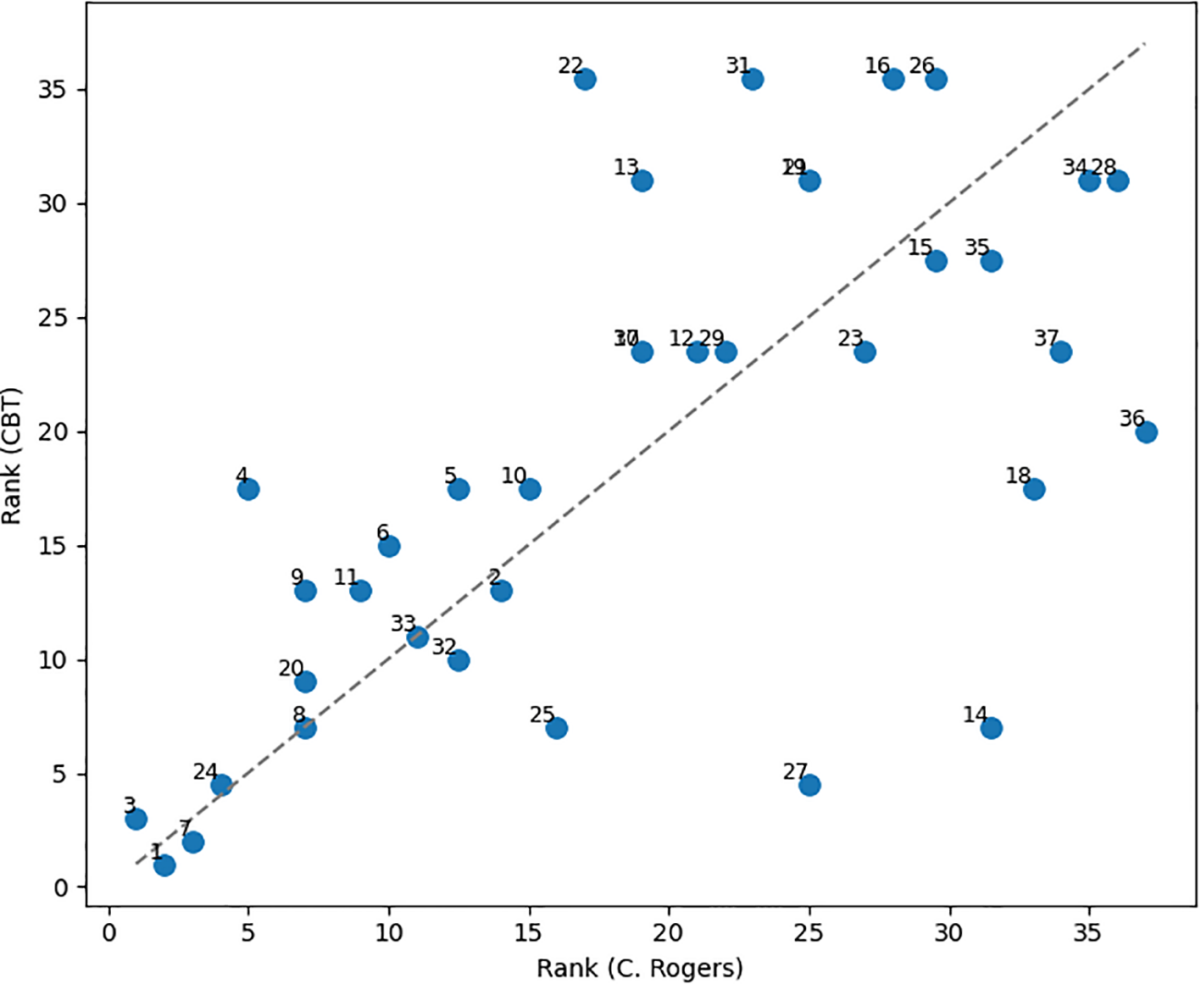
Ranked distribution of topics for modern CBT therapists and C. Rogers.

The Spearman correlation coefficient calculated for the full sample, in which the missing topics were assigned a rank of 35.5, revealed statistically significant correlation (r = 0.65, p < 0.0001). A visual representation of the rank distribution and initial data for the analyzed topic categories are presented in **Figure 3** and **Appendix D**, **Supplementary Table D.1**.

Analysis of both samples revealed distinct thematic differences. Specifically, topics such as 16 «Gender Roles and Relationships», 22 «Emotions of Crying and Tears», 26 «Pursuing Meaningful Personal Goals», and 31 «Therapy and Father Relationships», which were prominent in Rogers’ discourse, were entirely absent from the contemporary CBT corpus. One possible explanation for this disparity lies in the emphasis of Rogers’ therapeutic approach on deep emotional and existential exploration. For instance, a discussion of the client’s relationship with his/her father may serve as an entry point for understanding issues related to self-esteem. In contrast, CBT focuses on alleviating specific psychological symptoms (e.g., anxiety, depressive states) by modifying the client’s cognitions and behaviors.

It can be also noted that the topics 8 «Clarifying Meaning and Intent» and 33 «Revisiting the Past Together» exhibited identical rank assignments of 7 and 11, respectively. Among the topics differing by no more than one rank, the following can be highlighted: 1 «Time Up and Future Meetings», 2 «Complex Emotions Toward Him», 7 «See and Understanding Conversations», 24 «Possibilities and Potential Outcomes». With the exception of topic 2, these topics were consistently ranked within the top ten in both groups. Conversely, the most substantial divergence in rank was observed for topic 14 «Dynamics of Meaningful Relationships», ranked 31.5 in Carl Rogers’ sessions and 7 in CBT sessions. Additional discrepancies were found for topic 4 «Personal Growth and Decision-Making» (ranked 5 in Rogers, 17.5 in CBT) and topic 13 «Guilt and Self-Blame Dynamics» (ranked 19 in Rogers, 31 in CBT).

In summary, across all analyzed therapeutic sessions, core conversational elements such as session initiation, greetings, and clarification inquiries were consistently observed (topics 1 and 8). Topics with low prevalence also exhibited similar ranking patterns. For instance, topic 19 «Voices and Perception of Sound» ranked 25th in Rogers’ sessions and 31st in CBT, indicating uniformly low engagement with this theme across all samples. In contrast, topic 14 «Dynamics of Meaningful Relationships» showed minimal engagement in Rogers’ sessions but received substantial attention in CBT. Topic 13 «Guilt and Self-Blame Dynamics» appeared to be de-emphasized in CBT, while playing a more significant role in Rogers’ discourse. Similarly, topic 22 «Emotions of Crying and Tears» revealed a pronounced contrast between humanistic and cognitive-behavioral approaches. These discrepancies likely reflect the divergent therapeutic philosophies underlying the two approaches. Rogers’ client-centered therapy emphasizes emotional reflection and acceptance, treating emotions such as guilt as integral to personal growth. In contrast, CBT tends to view guilt as a cognitive distortion – a symptom to be corrected rather than deeply explored.

## 5 Limitations

This study has several limitations that should be considered when interpreting its findings. First, the sample size is restricted by the exclusive use of publicly available psychotherapy sessions. While this ensures transparency and replicability, it inevitably limits the volume and diversity of therapist-client interactions. A larger dataset would likely yield additional topics, providing a more comprehensive view of therapist discourse and therapeutic strategies.

Second, some analyzed sessions may have been recorded for educational or demonstrative purposes rather than as authentic therapeutic encounters. Such sessions, often created for training or public dissemination, may lack the depth and spontaneity of real-life therapy. Clients may temper their self-expression due to awareness of being recorded, potentially reducing the thematic richness of the data. Overcoming this limitation would require access to genuine therapy sessions, which raises significant ethical and confidentiality concerns.

Third, topic interpretation in this study was performed manually by experts to ensure semantic coherence and interpretability. While this yielded valuable qualitative insights, it limited the scalability and reproducibility of the approach. Future research could benefit from automated pipelines that identify and exclude low-interpretability topics. Advances in large language models may also support automated topic labeling and summarization, reducing subjective bias and labor demands.

## 6 Future work directions

Building on the current findings, future research could extend the analysis to include client speech alongside therapist remarks. Investigating the thematic content of client dialogue would provide a more balanced and comprehensive understanding of therapeutic discourse. This could reveal prevalent concerns, emotional states, and help-seeking patterns brought to therapy, thereby informing the design of training programs and improving therapist responsiveness to client needs.

Another promising direction is the temporal analysis of topic dynamics within individual sessions. By examining how topics emerge, shift, and resolve throughout the course of a session, researchers could gain deeper insights into the structure and effectiveness of therapeutic interventions. Such analysis could illuminate how therapists guide sessions, respond to client cues, and facilitate emotional processing over time.

In addition, future studies could explore greater automation of topic interpretation and filtering. As noted in the limitations, the current manual process, while valuable, is resource-intensive. Leveraging advances in large language models may enable automated labeling, summarization, and even emotional tagging of topics, thereby enhancing scalability and reducing subjective bias.

Finally, the integration of these methodologies could support the development of intelligent digital tools for therapists. By automatically identifying key topics and moments in therapy sessions, such systems could offer real-time suggestions for exploration, track progress, and assist with supervision and training. These tools hold the potential to augment clinical practice by providing timely insights, increasing therapeutic precision, and supporting data-informed decision-making in psychotherapy.

## 7 Conclusion

This study was conducted to analyze the topics of therapist remarks within a psychotherapeutic context. For this purpose, BERTopic, a powerful ML-based topic modeling technique, was applied to distinguish topics from unstructured dialogue of therapists with their clients within psychotherapy sessions. Topic modeling with a meticulous iterative process of optimizing topic structure based on expert assessment was applied to corpora of therapist remarks from both classical and modern therapeutic approaches, yielding 43 and 46 distinct topics, respectively. Analysis of semantic proximity within these topic sets identified prominent themes in the discourse of both therapist groups, likely reflecting core client concerns. These key topics include fear, anger, anxiety, familial and peer relationships, as well as issues related to development, self-esteem, and other psychological challenges.

Combining the topic structures of two corpora based on the analysis of cosine similarity between the topics allowed obtaining a merged topic structure, which was used for training the ML model able to analyze therapist remarks in a psychotherapeutic context, which demonstrated high accuracy in topic classification. Achieving an F1-score of 0.74 on the test set, it is possible to conclude that the proposed approach effectively automates the analysis of therapist communication topics. The ML model allows classifying 37 topic categories, which are unique as they emerge directly from natural human speech and relate to psychological context. They capture complex areas such as parent-child conflicts, addictive behavior, anxiety, fear, guilt, and depression – not merely as abstract topics but in terms of how they manifest and what underlying factors contribute to them.

The developed ML classifier was applied to a case study analyzing topic distribution in therapy sessions, specifically comparing Carl Rogers’ humanistic approach with sessions conducted using Cognitive Behavioral Therapy (CBT). Based on the outcomes of the ML classifier, a significant correlation between the two therapeutic approaches was revealed, with an observed Spearman rank correlation coefficient equal to r = 0.65 with p < 0.0001, which allowed highlighting similarities and differences in therapeutic directions. Fundamental conversational components like session initiation, greetings, and clarification remained consistent. However, significant divergences emerged in topics related to meaningful relationships, guilt, and self-blame issues, indicating shifts in client concerns and therapeutic emphases between the approaches. A particularly notable difference was observed in the topic discussing crying and tear emotions, which strikingly reflected Rogers’ emphasis on comprehensive emotional exploration versus CBT’s primary focus on cognitive restructuring.

These findings underscore the effectiveness of the developed ML classifier for practical application in a psychotherapeutic context, although the ML classifier has the potential to be applied to discussions of life situations more broadly. To facilitate further research and practical application, the proposed ML model is publicly available on the Hugging Face Hub.

## Data Availability

The original contributions presented in the study are included in the article/**Supplementary Material**. Further inquiries can be directed to the corresponding author.
