# Supplementary material for: Psychotherapist remarks’ ML classifier: insights from LLM and topic modeling application
Source: Front Psychiatry. 2025 Jul 25;16:1608163. doi: 10.3389/fpsyt.2025.1608163 (PMC12332746; doi:10.3389/fpsyt.2025.1608163)
Supplement: Supplementary file 1 [file SupplementaryFile1.docx]

Supplementary Material

Appendix A. Dataset Description

**Table A1.** Information on dataset employed in the manuscript

| **Video Name** | **Source Link** | **Channel Author** | **Therapy Direction** | **Group** | **Therapist** | **ID_client** |
| --- | --- | --- | --- | --- | --- | --- |
| CBT Role-Play – Complete Session – Social Anxiety Disorder – Part 1 | https://www.youtube.com/watch?v=gbBn8EzZx3w | Dr. Todd Grande | Cognitive Behavioral Therapy | modern | Todd Grande | 16 |
| CBT Role-Play – Complete Session – Social Anxiety Disorder – Part 4 | https://www.youtube.com/watch?v=LuuKIF4-F_Q | Dr. Todd Grande | Cognitive Behavioral Therapy | modern | Todd Grande | 16 |
| CBT Role-Play – Anxiety and Guilt Related to Balancing Home and Work | https://youtu.be/osROod3Hmpg?si=s7ZCqQXHRW99GgVB | Dr. Todd Grande | Cognitive Behavioral Therapy | modern | Todd Grande | 17 |
| CBT Role-Play - Behavioral Activation and Postpartum Depression | https://youtu.be/SNstOn6owcI?si=BZSIiDawi1Pdlw-Z | Dr. Todd Grande | Cognitive Behavioral Therapy | modern | Todd Grande | 18 |
| CBT Role-Play - Depressive Symptoms and Lack of Motivation | https://youtu.be/8aDFvvjC6XM?si=bP8vHY72uwKB_cRH | Dr. Todd Grande | Cognitive Behavioral Therapy | modern | Todd Grande | 19 |
| CBT Role-Play – Challenging Relationship with Family Member | https://www.youtube.com/watch?v=XbYGdo9lMsQ | Dr. Todd Grande | Cognitive Behavioral Therapy | modern | Todd Grande | 20 |
| CBT Role-Play – Managing Anger with Problems with Boyfriend & Coworkers | https://www.youtube.com/watch?v=W_tDRu67JnI&feature=youtu.be | Dr. Todd Grande | Cognitive Behavioral Therapy | modern | Todd Grande | 21 |
| CBT Role-Play – Loss of Hope in Career | https://www.youtube.com/watch?v=pllei-yDO8c | Dr. Todd Grande | Cognitive Behavioral Therapy | modern | Todd Grande | 22 |
| CBT Role-Play – Downward Arrow Technique; Test Anxiety | https://www.youtube.com/watch?v=Wx8F9uwQTnY | Dr. Todd Grande | Cognitive Behavioral Therapy | modern | Todd Grande | 21 |
| CBT Role Play – Catastrophizing and Decatastrophizing; Client Lost Job | https://www.youtube.com/watch?v=nanU4vR993I | Dr. Todd Grande | Cognitive Behavioral Therapy | modern | Todd Grande | 23 |
| CBT Role-Play – Complete Session – Low Self-Confidence at Work – Part 1 | https://www.youtube.com/watch?v=tq_yyMVax_c&list=PLeULT71Fr5DkbpzGToKlFwQJRT4HP7zmz&index=1 | Dr. Todd Grande | Cognitive Behavioral Therapy | modern | Todd Grande | 24 |
| CBT Role-Play – Complete Session – Low Self-Confidence at Work – Part 2 | https://www.youtube.com/watch?v=G40ywooLnns&list=PLeULT71Fr5DkbpzGToKlFwQJRT4HP7zmz&index=2 | Dr. Todd Grande | Cognitive Behavioral Therapy | modern | Todd Grande | 24 |
| CBT Role-Play – Complete Session – Low Self-Confidence at Work – Part 3 | https://www.youtube.com/watch?v=DcAqhHb5-hk&index=3&list=PLeULT71Fr5DkbpzGToKlFwQJRT4HP7zmz | Dr. Todd Grande | Cognitive Behavioral Therapy | modern | Todd Grande | 24 |
| CBT Role-Play – Complete Session – Low Self-Confidence at Work – Part 4 | https://www.youtube.com/watch?v=U6VzVpsqG40&list=PLeULT71Fr5DkbpzGToKlFwQJRT4HP7zmz&index=4 | Dr. Todd Grande | Cognitive Behavioral Therapy | modern | Todd Grande | 24 |
| CBT Role-Play – Complete Session – Low Self-Confidence at Work – Part 5 | https://www.youtube.com/watch?v=-tbKuW6k1cE&list=PLeULT71Fr5DkbpzGToKlFwQJRT4HP7zmz&index=5 | Dr. Todd Grande | Cognitive Behavioral Therapy | modern | Todd Grande | 24 |
| CBT Role-Play – Complete Session – Low Self-Confidence at Work – Part 6 | https://www.youtube.com/watch?v=KuHLL2AE-SE&list=PLeULT71Fr5DkbpzGToKlFwQJRT4HP7zmz&index=6 | Dr. Todd Grande | Cognitive Behavioral Therapy | modern | Todd Grande | 24 |
| CBT Role-Play – Complete Session – Low Self-Confidence at Work – Part 7 | https://www.youtube.com/watch?v=jS1KE3_Pqlc&list=PLeULT71Fr5DkbpzGToKlFwQJRT4HP7zmz&index=7 | Dr. Todd Grande | Cognitive Behavioral Therapy | modern | Todd Grande | 24 |
| CBT Role-Play – Complete Session – Low Self-Confidence at Work – Part 8 | https://www.youtube.com/watch?v=Ac0aZgha6Fk&list=PLeULT71Fr5DkbpzGToKlFwQJRT4HP7zmz&index=8 | Dr. Todd Grande | Cognitive Behavioral Therapy | modern | Todd Grande | 24 |
| CBT Role-Play – Complete Session – Low Self-Confidence at Work – Part 9 | https://www.youtube.com/watch?v=Q_kYJ63RUAA&list=PLeULT71Fr5DkbpzGToKlFwQJRT4HP7zmz&index=9 | Dr. Todd Grande | Cognitive Behavioral Therapy | modern | Todd Grande | 24 |
| CBT Role-Play – Complete Session – Low Self-Confidence at Work – Part 10 | https://www.youtube.com/watch?v=C4kdF-3btbg&list=PLeULT71Fr5DkbpzGToKlFwQJRT4HP7zmz&index=10 | Dr. Todd Grande | Cognitive Behavioral Therapy | modern | Todd Grande | 24 |
| CBT Role-Play – Complete Session – Low Self-Confidence at Work – Part 11 | https://www.youtube.com/watch?v=Bub8YLl1fUI&list=PLeULT71Fr5DkbpzGToKlFwQJRT4HP7zmz&index=11 | Dr. Todd Grande | Cognitive Behavioral Therapy | modern | Todd Grande | 24 |
| CBT Role-Play – Complete Session – Low Self-Confidence at Work – Part 12 | https://www.youtube.com/watch?v=rRze7Na1MXg&index=12&list=PLeULT71Fr5DkbpzGToKlFwQJRT4HP7zmz | Dr. Todd Grande | Cognitive Behavioral Therapy | modern | Todd Grande | 24 |
| CBT Role-Play – Cognitive Reframing an Experience of Emotional Abuse | https://www.youtube.com/watch?v=CdyJ0iB_k00 | Dr. Todd Grande | Cognitive Behavioral Therapy | modern | Todd Grande | 24 |
| CBT Role-Play – Managing Anger | https://www.youtube.com/watch?v=W_tDRu67JnI | Dr. Todd Grande | Cognitive Behavioral Therapy | modern | Todd Grande | 24 |
| DBT- Behavior Theory | https://www.youtube.com/watch?v=_gPcDRVALVo | Lisa Mansell | Dialectical Behavior Therapy | modern | Lisa Mansell | 28 |
| DBT- Client with Anger | https://www.youtube.com/watch?v=ofnnuwscA1Y | sarangoxio | Dialectical Behavior Therapy | modern | Therapist_3 | 30 |
| DBT- Client Affected with Anxiety; affecting daily functioning | https://www.youtube.com/watch?v=iBp75KNuLb8 | sarangoxio | Dialectical Behavior Therapy | modern | Therapist_3 | 30 |
| MI- Client with Smoking Cessation | https://www.youtube.com/watch?v=1jfH055byg4 | Behavioral Health & Wellness Program | Motivational Interviewing | modern | Therapist_4 | 31 |
| MI-Focus on Goals- High Risk Alcohol use Student | https://www.youtube.com/watch?v=_TwVa4utpII | [MerloLab](https://www.youtube.com/@MerloLab) | Motivational Interviewing | modern | Therapist_5 | 32 |
| MI- Part 1- Survivor of Intimate Partner Violence- Intake Session | https://www.youtube.com/watch?v=P3JUXQ4kkHs | Portland State University | Motivational Interviewing | modern | Dr. Stéphanie Wahab | 33 |
| IPT- Initial Phase for Client with Eating Disorder | https://www.youtube.com/watch?v=A1XJeciqyL8 | NEDA Study | Interpersonal Psychotherapy | modern | Therapist_6 | 34 |
| What a Dialectical Behavior Therapy (DBT) Session Looks Like | https://youtu.be/iQEurMdJtds?si=WMlM_kcVjQyPtapJ | MedCircle | Dialectical Behavior Therapy | modern | Dr. Judy | 35 |
| Here's What Humanistic Therapy Looks Like \| MedCircle x Dr Ramani | https://youtu.be/5DnJifOaHQ0?si=DcVlhC-9YsXc86Gr | MedCircle | Gestalt Therapy | modern | Dr. Ramani. | 35 |
| Session 10 with Abe from Cognitive Behavior Therapy: Basics and Beyond, 3rd Ed. | https://youtu.be/ikYCr-0GAfw?si=9t4V2DkvKr30Eeeg | Beck Institute for Cognitive Behavior Therapy | Cognitive Behavioral Therapy | modern | Dr. Beck | 36 |
| Session 2 with Abe from Cognitive Behavioral Therapy: Basics and Beyond, 3rd Ed. | https://youtu.be/ac5Jct33oUU?si=SeXODHEnz4mE9swY | Beck Institute for Cognitive Behavior Therapy | Cognitive Behavioral Therapy | modern | Dr. Beck | 36 |
| BPD Psych Interview \| Charlotte & Dr. Diamond | https://www.youtube.com/watch?v=8L1qhjQx73E&pp=ygUVcHN5Y2hvdGhlcmFweSBzZXNzaW9u | BorderlinerNotes | unknown | modern | Dr. Diamond | 37 |
| Transactional Analysis Psychotherapy--Free Child Ego state | https://www.youtube.com/watch?v=0K7KyXIWZ80&pp=ygUVcHN5Y2hvdGhlcmFweSBzZXNzaW9u | [Bob Cooke](https://www.youtube.com/@BobCooke) | Transactional Analysis | modern | [Bob Cooke](https://www.youtube.com/@BobCooke) | 38 |
| Psychiatric Interview: BPD (Borderline Personality Disorder) \| Part 1 \| Dr. Karen Jacob | https://youtu.be/MpMHl4Hby0I?si=2azaRDosSp-DnOEX | BorderlinerNotes | unknown | modern | Dr. Karen Jacob | 39 |
| Sample Psychoanalysis Session | https://youtu.be/tQZPd7e8lXw?si=haOyXjU7gFtGAvP3 | Mark Jones | Psychoanalysis | modern | Mark Jones | 40 |
| LIVE PSYCHOTHERAPY SESSION \| Helping a woman with some anger during COVID \| Albert Ellis Institute | https://youtu.be/9ewsD_RpybE?si=i52ECedghnB1QDyp | unknown | Rational Emotive Behavior Therapy | modern | Therapist_10 | 91 |
| LIVE Therapy Session with Dr. Ramani \| Part 2] | https://youtu.be/l2SNesXZoGM?si=IdjS8iSriHaRtVkY | MedCircle | unknown | modern | Dr. Ramani | 35 |
| Internal Family Systems [ IFS ] Therapy Demonstration with Dr Richard Schwartz | https://youtu.be/DGfrJu-Ck8o?si=crnAFB3b1HhBHGfl | [PCPSI](https://www.youtube.com/@PCPSITUBE) | Internal Family Systems Model | modern | Dr. Richard Schwartz i | 41 |
| Internal Family Systems Session 2022 | https://youtu.be/2-aAKVRIuSc?si=5eIUEtGkOGnJ8P8Q | Derek IFSCA | Internal Family Systems Model | modern | Derek Scott | 42 |
| What is Cognitive Behavioral Therapy?\| CBT Therapy Session For Anxiety | https://youtu.be/bM0BeeA8RdY?si=ILkRR5kOcYKAMMlw | Manicka Thomas \| The Social Work Success Path | Cognitive Behavioral Therapy | modern | Manicka Thomas | 43 |
| Demonstration of first counselling session with a 19 year old girl | https://youtu.be/Ssi7Rzvfc40?si=0YxnipTJcT8JeKt- | The Therapy Platform | unknown | modern | Sarah Poh | 44 |
| Person Centred Therapy Demonstration: A Contemporary Approach (No Commentary) | https://youtu.be/CMmzqH5gC5A?si=JEkgU7TX87TCIpzE | Mick Cooper | Person-Centered Therapy | modern | Mick Cooper | 45 |
| Counselling Session Demonstration: Julie | https://youtu.be/Md3rdQssxxE?si=9gIwjxf2elIwu7bU | Mick Cooper | Person-Centered Therapy | modern | Mick Cooper | 46 |
| Complete IFS Session | https://youtu.be/tqIhczaF9W8?si=kCsnM4TCdMnceWzd | Derek IFSCA | Internal Family Systems Model | modern | Derek Scott | 48 |
| LIVE ISTDP Therapy Session | https://youtu.be/XDlWSR7Ohck?si=Sj1BYtST93yCSwqi | MedCircle | Intensive short-term dynamic psychotherapy | modern | Dr. Kristy Lamb | 51 |
| Good Therapy Session. A Cardiothoracic Surgeon Struggles with Work/Life Balance. Kim Lampson, PhD | https://youtu.be/KKVTxHlvBsU?si=Dv4-D3_pXGqslg1F | Kim Lampson | Crisis Interventions | modern | Kim Lampson | 54 |
| Therapy Session: Good Counseling Skills. Hoarding Disorder. Kim Lampson, PhD | https://youtu.be/88qr91P1lsA?si=2GRYv8w9Ba0trTz0 | Kim Lampson | Crisis Interventions | modern | Kim Lampson | 56 |
| Therapy Session: Really Bad Counseling Skills 1 | https://youtu.be/FQOrzyBuAi8?si=J8OZTxlpIWMwzcK2 | Kim Lampson | Crisis Interventions | modern | Kim Lampson | 57 |
| N/A (text file) | Brodley, B.T., & Lietaer, G. (Eds.).(2006). Transcripts of Carl Rogers’ therapy sessions. Volume 12 Part 2 | N/A (text file) | Person-Centered Therapy | classic | Carl Rogers | 93 |
| Mock counselling session 1 | https://youtu.be/kdk71MRW8mk?si=AIs1ONUsh4ki2HAQ | [Amanda Sanzo](https://www.youtube.com/@amandasanzo4503) | unknown | modern | [Amanda Sanzo](https://www.youtube.com/@amandasanzo4503) | 58 |
| Mindfulness Therapy session on help for anxiety and depression medication reduction | https://youtu.be/0mrgqXoQI80?si=ge_UtWJ49iSel-CN | Bonnie Valles | Mindfulness therapy | modern | Bonnie Valles | 59 |
| UWS Clinical Mental Health Counseling Standardized Patient Full Session | https://youtu.be/wKgPxVC1GlU?si=KjnESdvofeY2evrz | Univ WesternStates | unknown | modern | Dr. Michelle Cox | 60 |
| Briana Mock Counseling Session | https://youtu.be/eQCyU2lqLH4?si=FqTQPT-Dqoi5o565 | Nick Wilson | unknown | modern | Therapist_7 | 61 |
| Eating Disorder Counselling Training Video - Example Session | https://youtu.be/LVpEsgvbr7k?si=tNvRSwHIhawcKv-R | Therapy Partners | unknown | modern | Therapist_8 | 62 |
| IFS Therapy DEMO #1 #IFStherapydemo | https://youtu.be/ql0gKiJKuRM?si=Yy-GlZlElpa6owtZ | Empowerment After Trauma | Internal Family Systems Model | modern | Chaya Feuerman | 63 |
| IFS Polarization Demo | https://youtu.be/Al7jnW-Z08w?si=5rvE_MRV9AoBdWqz | Internal Family Systems - IFS Institute | Internal Family Systems Model | modern | Dick Schwartz | 64 |
| IFS Therapy Demo #2 - Demystifying Our Parts #IFS Demo #MentalHealth #IFS #Traumatherapy | https://youtu.be/mobbK9v-W4U?si=dbFzrLb04bMshlwD | Empowerment After Trauma | Internal Family Systems Model | modern | Chaya Feuerman | 65 |
| IFS Unburdening Session | https://youtu.be/CV1Gfifj6cM?si=ShooHtGBIP1BsPou | Derek IFSCA | Internal Family Systems Model | modern | Derek Scott | 66 |
| A Demonstration of IFS session for Healing Anxiety - with Emily Kerpelman | https://www.youtube.com/live/HGCrH6KMh08?si=3wVUDpQkVtnr_mws | Spiritual Wanderlust | Internal Family Systems Model | modern | Therapist_9 | 67 |
| Demonstration of a Somatic Attachment Therapy Session | https://youtu.be/3yu1z_Gj1Cg?si=jtBYNjt2dcUGTQF1 | Karen Rachels, LMFT | Somatic Attachment Therapy | modern | Karen Rachels | 68 |
| Karen with Karla: #5 2021 SAT Video | https://youtu.be/Ajpwomi1xOs?si=dxJkUuq-a5rGRRpf | Karen Rachels, LMFT | Somatic Attachment Therapy | modern | Karen Rachels | 69 |
| EMDR Therapy Session | https://youtu.be/v4oZWQDQq1E?si=hK2YbRLyQ7kWmP3H | Kati Morton | Eye movement desensitization and reprocessing therapy | modern | Dr. Alexa Altman | 70 |
| Therapy Recordings - "Anna", Session 1, Part 1 | https://youtu.be/X47ODjcasUI?si=AJtMh-71FC0_2EpT | The Curious Mind | unknown | modern | Gabriel | 71 |
| Therapy Recordings - "Anna", session 1, part 2 | https://youtu.be/uTj33gQ4G38?si=sy570u9mq5_K8NGZ | The Curious Mind | unknown | modern | Gabriel | 71 |
| Therapy Recordings - "Anna", session 2, part 1 | https://youtu.be/GpwgwK5GCEA?si=28xU5aWh12alrbr1 | The Curious Mind | unknown | modern | Gabriel | 71 |
| Therapy Recordings - "Anna", session 2, part 2 | https://youtu.be/W3FqtEVcFFM?si=edRHyXSaTutikGrl | The Curious Mind | unknown | modern | Gabriel | 71 |
| Therapy Recordings - "Anna", session 3, part 1 | https://youtu.be/0IqkVLEsC3E?si=WLpUrWgxH_3JEQPX | The Curious Mind | unknown | modern | Gabriel | 71 |
| Therapy Recordings - "Anna", session 3, part 2 | https://youtu.be/zyc4on_c-58?si=Zv3ZRTIgDj0_VdvB | The Curious Mind | unknown | modern | Gabriel | 71 |
| EMDR Therapy Demonstration: Dissociative Considerations | https://youtu.be/5nOn-v1qoNI?si=66A2WgzwcBF6A4GB | Jamie Marich | Eye movement desensitization and reprocessing therapy | modern | Jamie Marich | 72 |
| Progressing without crashing - Everyday Alex Episode 002 | https://youtu.be/thONLD7Ygxs?si=w7CMAx_1d2mwl5bI | Alex Howard | unknown | modern | Alex Howard | 73 |
| Protecting progress on the recovery path \| Everyday Alex 90 \| Therapeutic Tuesday | https://youtu.be/Dil8iHlOSK8?si=mfIPu5Gpv-ILMqWy | Alex Howard | unknown | modern | Alex Howard | 73 |
| Understanding the roots of perfectionism - Everyday Alex 030 | https://youtu.be/rzKX2f6HJ30?si=6o4GfkHfTMI0TG2i | Alex Howard | unknown | modern | Alex Howard | 74 |
| Self-love calms the nervous system - Everyday Alex 009 | https://youtu.be/45ZJOku_Lfc?si=b3y-xXg6AZdLvkrL | Alex Howard | unknown | modern | Alex Howard | 75 |
| Working with anxiety in the body - Everyday Alex 037 - Everyday Alex | https://youtu.be/zYRmgerjHfY?si=MyL-NaoNGfHwa6cz | Alex Howard | unknown | modern | Alex Howard | 75 |
| Your body is trying to protect you \| Everyday Alex 076 | https://youtu.be/RYS_uswYoVU?si=wl-hZb4oRSt_Edqr | Alex Howard | unknown | modern | Alex Howard | 75 |
| The power of routine - Everyday Alex 044 | https://youtu.be/ocJixNv5BaY?si=JXtxkhXlQj_P6Hrn | Alex Howard | unknown | modern | Alex Howard | 76 |
| Why isn't my energy coming back? \| Everyday Alex 071 | https://youtu.be/m_go88HWlJY?si=Dgxns1O9AnhdjGKc | Alex Howard | unknown | modern | Alex Howard | 77 |
| Understanding why the body shuts down \| Everyday Alex 066 \| Therapeutic Tuesdays | https://youtu.be/cLg9ATrMvww?si=ZlFHfy5K--kcWxBE | Alex Howard | unknown | modern | Alex Howard | 78 |
| How to recover whilst managing external stress \| Everyday Alex 085 | https://youtu.be/omw8JZM0Zxc?si=ZOoO6yu8mPUiCvC3 | Alex Howard | unknown | modern | Alex Howard | 79 |
| Connection feeds connection - Everyday Alex 023 | https://youtu.be/BTgtzhSIVRg?si=eXWbGQorWb_EklPT | Alex Howard | unknown | modern | Alex Howard | 80 |
| Your body knows how much it should be doing \| Everyday Alex 056 | https://youtu.be/RxUqn4_el1w?si=E0RCK6aRREAnRFYK | Alex Howard | unknown | modern | Alex Howard | 81 |
| How much activity should you do with CFS recovery? \| Everyday Alex 100 \| Therapeutic Tuesday | https://youtu.be/I1fyt5qmkoI?si=pjjS3t12moa3X0cM | Alex Howard | unknown | modern | Alex Howard | 78 |
| Why am I not improving? \| Everyday Alex 105 \| Therapeutic Tuesday | https://youtu.be/RjTOFTkojo0?si=FZS3EKIkwbu-Ihet | Alex Howard | unknown | modern | Alex Howard | 82 |
| Staying in contact with yourself \| Everyday Alex 051 | https://youtu.be/L4bgvbJ5HeI?si=exUspT7kRWg0EnhK | Alex Howard | unknown | modern | Alex Howard | 83 |
| Managing recovery around other people \| Everyday Alex 115 | https://youtu.be/hwm4XppX_y0?si=p3f7ECEQ9TPAg_B1 | Alex Howard | unknown | modern | Alex Howard | 84 |
| Listen to your body - Everyday Alex 016 | https://youtu.be/3Pxl29iMCrc?si=RimeF-uhppNvt7Hb | Alex Howard | unknown | modern | Alex Howard | 85 |
| Dealing with overwhelm \| Everyday Alex 110 \| Therapeutic Tuesday | https://youtu.be/ga0yDEltrBk?si=13G9hY99e_mNssmx | Alex Howard | unknown | modern | Alex Howard | 86 |
| Why diagnosis matters \| Everyday Alex 061 | https://youtu.be/CPBGzLzvIBw?si=WAk1XQrfuAB85nWL | Alex Howard | unknown | modern | Alex Howard | 87 |
| Managing recovery in a relationship \| Everyday Alex 095 \| Therapeutic Tuesday | https://youtu.be/F9Id90uOW6U?si=jyq6MPPbI27dvLE2 | Alex Howard | unknown | modern | Alex Howard | 88 |
| Recorded Session 1- Counseling Lenny | https://youtu.be/LqzwjsQbZTQ?si=gQuwVeqC6W5FaKlj | Debbie Magana | Motivational interviewing | modern | Debbie Magana | 89 |
| Counseling Session 2 | https://youtu.be/qv6L3i_eQgE?si=S8bTSSinHgSdygGJ | Debbie Magana | Motivational interviewing | modern | Debbie Magana | 89 |
| Therapy Recordings - "Hanni", session 1 | https://youtu.be/6bryrnVWGx8?si=7g5G4lgw9ftxskNB | The Curious Mind | unknown | modern | Gabriel | 90 |
| Therapy Recordings - "Hanni", session 2 | https://youtu.be/My9Lmsh1Sv0?si=ljv0DEX-1U0SIaYL | The Curious Mind | unknown | modern | Gabriel | 90 |
| Therapy Recordings - "Hanni", session 3 | https://youtu.be/pQx4f2u9R6E?si=EbWGc1gFpzJ-09jq | The Curious Mind | unknown | modern | Gabriel | 90 |
| 2. Gloria Films. Fritz Perls and Gloria (англ. и рус. субтитры) | https://youtu.be/Ipcz-ufH3F0?si=ZlxtQvMMNjFW7pkH | Elena Tikhomirova | Gestalt Therapy | classic | Fritz Perls | 1 |
| 3. Albert Ellis and Gloria Counselling 1965 Full Session Rational Emotive Therapy | https://youtu.be/LVdZdVCyAk8?si=LtnQReMAWXgA2LqH | Elena Tikhomirova | Rational Emotive Behavior Therapy | classic | Albert Ellis | 1 |
| N/A (text file) | Brodley, B.T., & Lietaer, G. (Eds.).(2006). Transcripts of Carl Rogers’ therapy sessions. Volume 10 | N/A (text file) | Person-Centered Therapy | classic | Carl Rogers | 2 |
| N/A (text file) | Brodley, B.T., & Lietaer, G. (Eds.).(2006). Transcripts of Carl Rogers’ therapy sessions. Volume 11 | N/A (text file) | Person-Centered Therapy | classic | Carl Rogers | 3 |
| N/A (text file) | Brodley, B.T., & Lietaer, G. (Eds.).(2006). Transcripts of Carl Rogers’ therapy sessions. Volume 12 Part 1 | N/A (text file) | Person-Centered Therapy | classic | Carl Rogers | 1 |
| N/A (text file) | Brodley, B.T., & Lietaer, G. (Eds.).(2006). Transcripts of Carl Rogers’ therapy sessions. Volume 13 Part 1 | N/A (text file) | Person-Centered Therapy | classic | Carl Rogers | 4 |
| N/A (text file) | Brodley, B.T., & Lietaer, G. (Eds.).(2006). Transcripts of Carl Rogers’ therapy sessions. Volume 14 | N/A (text file) | Person-Centered Therapy | classic | Carl Rogers | 5 |
| N/A (text file) | Brodley, B.T., & Lietaer, G. (Eds.).(2006). Transcripts of Carl Rogers’ therapy sessions. Volume 15 | N/A (text file) | Person-Centered Therapy | classic | Carl Rogers | 6 |
| N/A (text file) | Brodley, B.T., & Lietaer, G. (Eds.).(2006). Transcripts of Carl Rogers’ therapy sessions. Volume 16 Part 1 | N/A (text file) | Person-Centered Therapy | classic | Carl Rogers | 7 |
| N/A (text file) | Brodley, B.T., & Lietaer, G. (Eds.).(2006). Transcripts of Carl Rogers’ therapy sessions. Volume 17 | N/A (text file) | Person-Centered Therapy | classic | Carl Rogers | 8 |
| N/A (text file) | Brodley, B.T., & Lietaer, G. (Eds.).(2006). Transcripts of Carl Rogers’ therapy sessions. Volume 2 | N/A (text file) | Person-Centered Therapy | classic | Carl Rogers | 9 |
| N/A (text file) | Brodley, B.T., & Lietaer, G. (Eds.).(2006). Transcripts of Carl Rogers’ therapy sessions. Volume 3 | N/A (text file) | Person-Centered Therapy | classic | Carl Rogers | 10 |
| N/A (text file) | Brodley, B.T., & Lietaer, G. (Eds.).(2006). Transcripts of Carl Rogers’ therapy sessions. Volume 4 | N/A (text file) | Person-Centered Therapy | classic | Carl Rogers | 11 |
| N/A (text file) | Brodley, B.T., & Lietaer, G. (Eds.).(2006). Transcripts of Carl Rogers’ therapy sessions. Volume 5 | N/A (text file) | Person-Centered Therapy | classic | Carl Rogers | 12 |
| N/A (text file) | Brodley, B.T., & Lietaer, G. (Eds.).(2006). Transcripts of Carl Rogers’ therapy sessions. Volume 6 | N/A (text file) | Person-Centered Therapy | classic | Carl Rogers | 13 |
| N/A (text file) | Brodley, B.T., & Lietaer, G. (Eds.).(2006). Transcripts of Carl Rogers’ therapy sessions. Volume 7 | N/A (text file) | Person-Centered Therapy | classic | Carl Rogers | 14 |
| N/A (text file) | Brodley, B.T., & Lietaer, G. (Eds.).(2006). Transcripts of Carl Rogers’ therapy sessions. Volume 8 | N/A (text file) | Person-Centered Therapy | classic | Carl Rogers | 15 |
| N/A (text file) | Brodley, B.T., & Lietaer, G. (Eds.).(2006). Transcripts of Carl Rogers’ therapy sessions. Volume 9 | N/A (text file) | Person-Centered Therapy | classic | Carl Rogers | 15 |
| N/A (text file) | Brodley, B.T., & Lietaer, G. (Eds.).(2006). Transcripts of Carl Rogers’ therapy sessions. Volume 13 Part 2 | N/A (text file) | Person-Centered Therapy | classic | Carl Rogers | 94 |
| N/A (text file) | Brodley, B.T., & Lietaer, G. (Eds.).(2006). Transcripts of Carl Rogers’ therapy sessions. Volume 13 Part 3 | N/A (text file) | Person-Centered Therapy | classic | Carl Rogers | 95 |
| N/A (text file) | Brodley, B.T., & Lietaer, G. (Eds.).(2006). Transcripts of Carl Rogers’ therapy sessions. Volume 13 Part 4 | N/A (text file) | Person-Centered Therapy | classic | Carl Rogers | 96 |
| N/A (text file) | Brodley, B.T., & Lietaer, G. (Eds.).(2006). Transcripts of Carl Rogers’ therapy sessions. Volume 16 Part 2 | N/A (text file) | Person-Centered Therapy | classic | Carl Rogers | 97 |
| N/A (text file) | Brodley, B.T., & Lietaer, G. (Eds.).(2006). Transcripts of Carl Rogers’ therapy sessions. Volume 16 Part 3 | N/A (text file) | Person-Centered Therapy | classic | Carl Rogers | 98 |
| N/A (text file) | Brodley, B.T., & Lietaer, G. (Eds.).(2006). Transcripts of Carl Rogers’ therapy sessions. Volume 16 Part 4 | N/A (text file) | Person-Centered Therapy | classic | Carl Rogers | 99 |
