# Supplementary material for: Psychotherapist remarks’ ML classifier: insights from LLM and topic modeling application
Source: Front Psychiatry. 2025 Jul 25;16:1608163. doi: 10.3389/fpsyt.2025.1608163 (PMC12332746; doi:10.3389/fpsyt.2025.1608163)
Supplement: Supplementary file 2 [file SupplementaryFile2.docx]

Supplementary Material

Appendix B. Descriptions of the Identified Topics for Classical and Modern Therapists


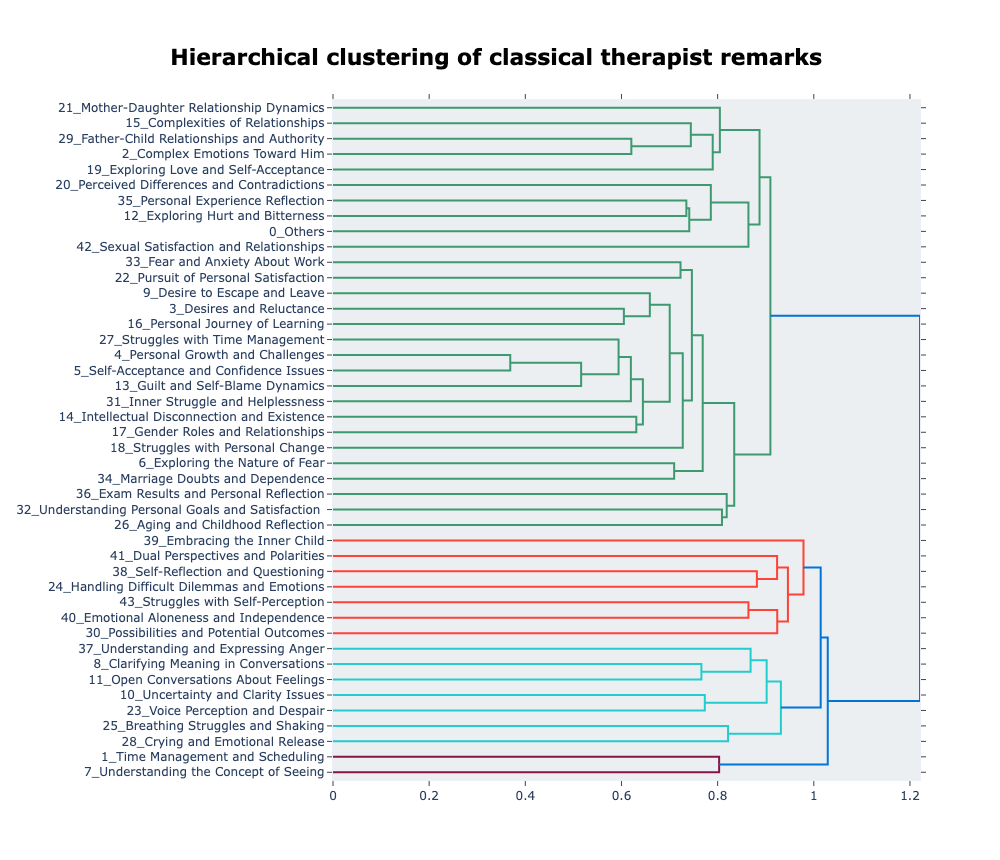


**Figure B1.** Results of hierarchical cluster analysis of classical therapist remarks

**Table B1.** Topics of Classical Therapists

| **Topic (Number of Objects)** | **Top 10 Keywords** | **Topic Description** |
| --- | --- | --- |
| 0 Others (3827) | 'mhm', 'mhm mhm', 'her', 'she', 'that', 'the', 'okay', 'mhm that', 'not', 'it' | This category includes various topics, as well as paralinguistic expressions, which were removed due to their insufficient depth for analysis. |
| 1 Time Management and Scheduling. (355) | 'we', 'time', 'our', 'our time', 'week', 'next', 'see', 'monday', 'friday', 'stop' | The topic includes both the therapist’s (‘T’) statements regarding the management of the therapy session's duration («I see our time is up») and remarks directed at planning a new meeting («Shall we make it next week at the same time?»). |
| 2 Complex Emotions Toward Him (302) | 'he', 'him', 'his', 'that he', 'you', 'that', 'to', 'and', 'feel', 'not' | T examines the client’s (‘C’) relationship with an abstract male figure («You feel he has a dark critic inside of him», «There are certain things you respect him for, but that doesn’t, uh, alter the fact that you definitely hate him and don't love him»). |
| 3 Desires and Reluctance (274) | 'do', 'not', 'do not', 'want', 'not want', 'you do', 'want to', 'you', 'to', 'anything' | T identifies desirable and undesirable scenarios for C («You don’t want to make it any darker than it is», «You don’t want to starve and you don’t want to cross the Alps»). |
| 4 Personal Growth and Challenges (215) | 'you', 'to', 'that', 'the', 'of', 'in', 'that you', 'and', 'it', 'feel' | T addresses C’s lack of clarity regarding his/her life direction («You don't know at all where you want to, what direction you want to move or what you want to do»). T also interprets C’s indecision in choosing a life path («You feel that on the one hand you're not living up to all the things you really should be doing, but on the other hand, you feel that it's more realistic to grow gradually into that») and highlights his/her progress in personal growth («I feel that you’ve made a good deal of progress inside yourself»). |
| 5 Self-Acceptance and Confidence Issues (212) | 'you', 'that', 'in', 'feel', 'you feel', 'to', 'confidence', 'them', 'your', 'the' | T explores C’s confidence and lack of confidence in himself/herself. For instance, T notes C’s confidence in his/her feelings («You feel kind of a confidence in your own feelings»), discusses potential reasons for C’s sense of inadequacy («You feel that something should have turned up to give you that confidence in yourself»), and analyzes aspects of C’s self-perception in the context of how he/she is perceived by others («You feel that you are living by the standards others have and what they think of you and so on, even though more deeply you know that you can't possibly have happiness that way»). |
| 6 Exploring the Nature of Fear (200) | 'fear', 'afraid', 'the fear', 'of', 'of fear', 'the', 'fear of', 'afraid of', 'you', 'risk' | T addresses the nature of C’s fear, including various forms of fear («They nevertheless are fears deep inside, and the biggest fear of all is the fear of being trapped, in so many different ways», «So that's where you fear is, a fear of a relationship with a man», «It really wasn’t the fear of death; that you can accept», «Fear of choking, fear of convulsions?», «It seems to me that you're saying, ahh, the fear, the fears grow stronger, as time goes by, both of marriage and of children and of commitment, as well as the fear of aging... that it seems a package of fears»). T also attempts to normalize C’s fear («The fear comes and goes», «To know that you are not alone in that fear, the others have the same kind of fear») and interprets his/her anxious state («The more clear our feeling of fear that exists in us here is blocked off close to you, that then somehow it develops into this more nameless, more indescribable kind of fear that is anxiety»). |
| 7 Understanding the Concept of Seeing (175) | 'see', 'see see', 'mhm see', 'see mhm', 'let', 'let is', 'get that', 'get', 'is see', 'see if' | T demonstrates his/her readiness to address the problematic situation («Let's see», «But let's just look at that») and shows understanding in response to C’s remarks («I see»), thereby supporting the dynamics of the therapeutic process. |
| 8 Clarifying Meaning in Conversations (157) | 'what you', 'saying', 'that what', 'what', 'are saying', 'say', 'mean', 'meaning', 'you mean', 'to say' | The topic contains T’s statements aimed at clarifying what C has said («Is that what you mean?», «Is that what you're saying?»). |
| 9 Desire to Escape and Leave (118) | 'away', 'away from', 'leave', 'it', 'you', 'from', 'to', 'out', 'get', 'get away' | T explores both the C’s desire to escape the situation he/she finds himself/herself in («Seems as though the thing that is stirring within you is more 'I want to get away, be out on my own, be free'») and the difficulties that accompany this («And you know what a struggle it was to move away from that», «Just felt that there was a need to get away and you couldn't explain it to others; you just couldn't»). |
| 10 Uncertainty and Clarity Issues (105) | 'not know', 'not quite', 'sure', 'not', 'quite', 'am not', 'do not', 'know', 'quite sure', 'am' | T acknowledges that he/she do not fully understand C or his/her situation («I'm not quite sure»), and also notes the uncertainty in C’s words («You're not quite sure why»). |
| 11 Open Conversations About Feelings (99) | 'tell me', 'tell', 'me', 'talk', 'appreciate', 'to talk', 'what', 'about', 'to tell', 'to' | The topic contains T’s statements intended to begin the therapy session with C («Now, what I would like would be for you to tell me anything you’re willing to tell me about yourself and your situation»), as well as other requests for information from T to C («Tell me, what's your first name?», «Can you tell me any more of your thinking about it?»). |
| 12 Exploring Hurt and Bitterness (85) | 'hurt', 'pain', 'bitterness', 'the hurt', 'of', 'and', 'it', 'hurt and', 'the bitterness', 'the' | T explores the area of C’s emotional pain and bitterness. Specifically, T identifies the presence of painful feelings («It looks like you’re feeling some of that hurt right now»), analyzes their causes («That’s the way you have been hurt»), emphasizes the importance of reflection on pain («It's been so good to get out both the hurt and the bitterness where you can look at them»), and explains the different facets of pain to C («There’s a lot of pain there – pain about the past and pain about the present»). |
| 13 Guilt and Self-Blame Dynamics (80) | 'blame', 'for', 'you', 'feel', 'to blame', 'that', 'yourself', 'guilt', 'you feel', 'guilty' | T addresses C’s sense of guilt («You feel guilty about what you haven't done, and it gives you more reason for avoiding the people»), tries to explain the origins of this feeling («You don’t like to blame your school or your family, but still you feel that to some extent your family was responsible»), and explores C’s self-blame («That sounds like not only your family looks down on it or something but that you scold yourself for it too»). |
| 14 Intellectual Disconnection and Existence (79) | 'world', 'intellectual', 'the world', 'the', 'it', 'you', 'in', 'not', 'universe', 'of' | T explores C’s relationship with the world around him/her («You feel at real odds somehow with the world in general», «You don't like that feeling that the whole world's wrong»). T also legitimizes C’s value orientations («I don't know whether it's as a philosopher, but I certainly would agree with you that, in situations of this kind, I don't think there is any proof that could be advanced that would prove one set of values rather than the other») and challenges excessive rationalization («So maybe the intellectual understanding isn't as important as you thought it was»). |
| 15 Complexities of Relationships (78) | 'relationship', 'the relationship', 'relationships', 'relationship that', 'relationship you', 'that relationship', 'of', 'the', 'that', 'like' | T analyzes various aspects of C’s relationships with others («You know that it’s a difficult relationship, and you’d like to preserve it, but it’s going to take a great deal of energy on your part to hold that relationship together»), including possible strategies for improvement («It doesn't spoil the relationship to say ‘No’»). |
| 16 Personal Journey of Learning (71) | 'learn', 'to learn', 'learning', 'you', 'school', 'to', 'and', 'your', 'the', 'what' | T explores C’s desires in the areas of learning and education («You feel a real desire to try to learn some of this for what you would like to learn from it, not in terms of meeting some future examination demand or any other future demand», «You feel that you really like to achieve and learn a lot, but you just, you're just not doing it at present»). |
| 17 Gender Roles and Relationships (68) | 'women', 'woman', 'men', 'feminine', 'you', 'man', 'role', 'in', 'of', 'that' | T examines C’s gender roles. Specifically, T interprets C’s issues («So, it's really a big problem for you, you are feminine and you like to be feminine and you're seen as feminine and reacted to in that way, and then you think, ‘Oh my God!’», «When you feel insecure about the better sexual relationship, it might be because it hits you kind of hard that that stresses the fact that you are a woman with a woman's needs») and notes C’s masculinity («You see both elements of yourself pretty sharply, where on the one hand you may be more of a woman than you think you are, and on the other hand you have pretty masculine interests along some lines»). |
| 18 Struggles with Personal Change (67) | 'change', 'to change', 'changes', 'past', 'you', 'to', 'change in', 'the', 'in the', 'in' | T addresses C’s personal changes: touching on aspects such as C’s procrastination («You think to make a change you ought to change right now; on the other hand, you think that maybe it would have been simpler to delay and change next quarter») and interpreting the difficulties with change that C experiences («But you're just very much aware of a loosening type of change taking place, and intellectually when you can stand off and look at it to see, if...you knew exactly what made the wheels go round, hm?», «You tend to dwell a little more on what you didn't do in the past, rather than on what might be done right now or in the future»). |
| 19 Exploring Love and Self-Acceptance (63) | 'love', 'person', 'somebody', 'to', 'want', 'you meet', 'yourself', 'now let', 'meet', 'affection' | T analyzes C’s romantic relationships and self-acceptance. For instance, T normalizes C’s feelings regarding unrequited love («So, it hurt not to get that love back») or explores the facets of love («That there is a need for a love that isn't possessive and that just exists»). |
| 20 Perceived Differences and Contradictions (63) | 'different', 'something', 'is something', 'contradictory', 'it', 'is', 'difference', 'that', 'different from', 'discrepancy' | T addresses inconsistency between people's words and behavior («It is just an absurdly contradictory situation, in which we people say one thing and do the complete opposite»). T also explores differences among individuals («They somehow seem to belong together and yet they came out quite differently»), including differences in personal values («It sounds as though it isn’t any one big difference in values but a number of little things that pile up and pile up until you think, ‘Oh, the difference is too great’»). |
| 21 Mother-Daughter Relationship Dynamics (61) | 'mother', 'your mother', 'your', 'mother and', 'my mother', 'my', 'to', 'you', 'her', 'that' | T analyzes C’s relationship with her mother («It makes you feel as though…you are a mouse in your relationship to your mother») and notes C’s personality traits that are similar to those of her mother («So, you’re saying your mother was the model for this organizing self»). |
| 22 Pursuit of Personal Satisfaction (56) | 'satisfaction', 'satisfactions', 'happiness', 'the satisfactions', 'the', 'more', 'of', 'you', 'good', 'of satisfaction' | T addresses C’s sense of inner satisfaction («It seems more that the sense of satisfaction is a sort of total process of change that you feel and you move with it») and seeks to offer C an alternative perspective on the possibility of achieving this state («You realize there could be some satisfaction in doing something different from what you might be told»). |
| 23 Voice Perception and Despair (52) | 'voice', 'hear', 'the voice', 'did not', 'heard', 'did', 'not hear', 'hear you', 'not', 'quite' | T metaphorically addresses C’s inner voice («I guess another puzzling thing about it is that you feel that you hadn’t heard the voice for quite a while, why, why would it return?», «And was it the voice that said, ‘If you are feeling desperate...’»), also noting the despair in C’s tone («Oh, the voice sounded kind of desperate»). This topic also includes T’s statements about the volume of C’s voice («I didn't quite hear what you said»). |
| 24 Handling Difficult Dilemmas and Emotions (52) | 'too', 'too much', 'too strongly', 'hard', 'tough', 'putting it', 'dilemma', 'difficult', 'strongly', 'it' | T addresses the dilemmas and difficulties in C’s life, possibly with the intention of normalizing his/her emotions regarding the situation he/she finds himself/herself in («It is a real dilemma», «For you, you feel it's almost too much, is that it?»). |
| 25 Breathing Struggles and Shaking (51) | 'shaking', 'can not', 'throat', 'choking', 'head', 'your', 'your throat', 'choke', 'can', 'place of' | T interprets the cause of C’s trembling («Your shaking is really fear») and also notes other physiological manifestations in C’s behavior («You can't breathe», «Your head's spinning?»). This topic also includes C’s smoking habits («It doesn't keep you from going back to smoking», «I don’t smoke, but if you’ve got cigarettes, feel free to light up one»). |
| 26 Aging and Childhood Reflection (50) | 'childhood', 'childish', 'age', 'old', 'eighteen', 'young', 'you', 'and', 'childhood and', 'your childhood' | T explores C’s world through the lens of childhood and aging («You may behave in a certain way, and then only later realize that that was a step from childhood into adolescence»). T also addresses C’s fear of growing old («Can you tell me a little bit more about your fear that you have of aging?»). |
| 27 Struggles with Time Management (49) | 'time', 'your time', 'you', 'to', 'that', 'not', 'just', 'the', 'feel', 'you feel' | T addresses C’s experience of life's meaningfulness («In other words, it isn't just a question of filling in your time...»), his/her sense of time scarcity («You feel that right now you really waste a great deal of time that you actually can't afford to waste»), and aspects of time management («You've always felt that there was hardly enough time and you've had to utilize every scrap of it to get your things done»). |
| 28 Crying and Emotional Release (48) | 'cry', 'tears', 'the tears', 'if', 'crying', 'brings', 'would', 'weeping', 'cry it', 'brings the' | T discusses sensitive topics with C that may evoke tears («It almost brings tears to your eyes, doesn't it?»), also noting C’s tears («There are tears now») and normalizing his/her feelings about them, thereby expressing empathy («Yes, you could cry»). |
| 29 Father-Child Relationships and Authority (47) | 'father', 'your', 'your father', 'dad', 'with', 'with your', 'he', 'stepfather', 'relationship', 'brother' | T analyzes various facets of C’s relationship with his/her father or stepfather («That things went somewhat better between you and your father», «Just like your father»). |
| 30 Possibilities and Potential Outcomes (47) | 'possible', 'might', 'that would', 'would', 'would that', 'maybe', 'that', 'perhaps', 'maybe that', 'possibility' | T draws C’s attention to various possible outcomes («It’s one possible option at any rate», «Yes, probably that would be another possible outcome»). |
| 31 Inner Struggle and Helplessness (46) | 'struggle', 'you', 'it', 'you feel', 'pretty', 'feel', 'that', 'and', 'situation', 'to' | T explores C’s inner struggle («You're inclined to feel that war situation or no war situation, the struggle is pretty much within you, after all»), his/her sense of helplessness («You feel that it's really the back of the coming in is the fact that you feel helpless to do anything about it»), potential strategies for overcoming this feeling («In other words, when you begin to feel hopeless then it seems so necessary to distract yourself from yourself»), and the possibility of external support («It's pretty deeply annoying to get into the conflicts and then not be sure which way to go and wish like hell somebody would give a little push»). |
| 32 Understanding Personal Goals and Satisfaction (45) | 'goal', 'goals', 'the goal', 'reach', 'you', 'the', 'and', 'that', 'satisfied', 'achieve' | T examines C’s personal goals. T analyzes the nature of these goals («In other words, the goal as nearly as you can formulate it is some kind of fusion between the things intellectually you know you want and something pretty deep in you that doesn't lend itself easily to words, or labels or...»), notes the connection between goal achievement and self-knowledge («Whereas the kind of goal you want to reach probably can be more easily achieved when you understand some aspects of yourself»), assesses C’s progress in reaching personal goals («It’s just the gradual realization that you are not as far toward the goal as you hoped»), and identifies the necessary conditions for C to achieve these goals («You expected to reach the goal without the work or struggle that went in between»). |
| 33 Fear and Anxiety About Work (36) | 'job', 'work', 'the', 'to work', 'you', 'get job', 'job or', 'do', 'the job', 'of work' | T discusses C’s negative emotions related to work («You feel as though staying in the job situation may really bring a blow-up on your part», «That is, sort of asking yourself, why the hell should I be fearful of or overwhelmed by the notion of a job?»). |
| 34 Marriage Doubts and Dependence (34) | 'marriage', 'husband', 'your husband', 'married', 'your', 'your marriage', 'on', 'trapped', 'you', 'the' | T examines C’s relationship with his/her life partner, including aspects such as C’s feelings in marriage («You’re not happy with your husband and he’s not happy with you?») and fear of commitment («There is a fear of commitment, and a fear of having children. And a feeling that in marriage you don't want to give up your identity»). T also presents for consideration C’s hidden reasons for entering into marriage («Perhaps one of the things you looked forward to in marriage was that there would be a situation and a person on which you could basically depend»). |
| 35 Personal Experience Reflection (30) | 'experience', 'own experience', 'experienced', 'about', 'it', 'situation', 'about it', 'that', 'experience that', 'you have' | T encourages C by highlighting his/her positive life experiences («It really was pretty swell the way you handled that fraternity situation on the photographic job») and also refers to his/her own experience («I’ll simply speak from my own experience, and I’m not sure that it applies to you»). |
| 36 Exam Results and Personal Reflection (30) | 'test', 'exams', 'exam', 'the exam', 'the results', 'results', 'the', 'tell you', 'do about', 'what to' | T discusses both the exam situation with C («At least you're not sorry for having taken the exams and having made that decision») and educates C about existing psychological tests («And the Rorschach test you probably know – that's the one with the ink blots»), providing their results («Well, I can show you the results of the tests»). |
| 37 Understanding and Expressing Anger (27) | 'anger', 'angry', 'rage', 'anger and', 'and', 'there', 'you could', 'explaining', 'angry at', 'the anger' | T explores the nature of C’s anger. Specifically, T notes anger in C’s speech («I can hear the anger in there»), legitimizes C’s desire to feel anger («If you feel like being angry, you can be angry»), and discusses anger management strategies («So, when you meet anger, whether in men or women, you tend to placate it if possible»). |
| 38 Self-Reflection and Questioning (25) | 'question', 'the question', 'asking', 'asking yourself', 'raising', 'question you', 'yourself', 'question that', 'yourself so', 'you are' | T notes C’s self-reflection («It sounds like you’re asking yourself that, as well as me», «So, you’re raising the whole question, ‘Is it really productive to discuss so much about these value systems?’») and the frequent repetition of the same questions by C to himself/herself («That's the question you keep asking yourself»). |
| 39 Embracing the Inner Child (24) | 'girl', 'little girl', 'little', 'naughty little', 'naughty', 'daughter', 'daughter you', 'as my', 'the little', 'that little' | T metaphorically and with a touch of irony addresses C, emphasizing her attention on her inner self («You know that little girl is inside of you», «The naughty little girl can get away with things») and also addressing the nature of growing up («The little girl, the little girl will grow up if you care enough for her»). |
| 40 Emotional Aloneness and Independence (24) | 'alone', 'let me', 'alone you', 'aloneness', 'to alone', 'lonely', 'because do', 'with you', 'independent', 'as though' | T examines C’s sense of loneliness («You felt very much alone emotionally», «It’s as though you're in some way sort of responsible for your loneliness»). |
| 41 Dual Perspectives and Polarities (23) | 'two', 'sides', 'both', 'one', 'so there', 'two sides', 'split', 'both sides', 'angle of', 'are two' | T addresses the duality of C’s nature («Between the two selves») and desires («You want both of those directions»), exploring his/her polarization («But I get a sense of two poles there»). |
| 42 Sexual Satisfaction and Relationships (21) | 'sexual', 'sex', 'life', 'normal', 'satisfaction', 'that the', 'that', 'of', 'maybe', 'and' | T analyzes various aspects of C’s sexuality, including C’s satisfaction with his/here sexual life («If you couldn't find sexual satisfaction, that would be a failure of a very deep sort»), psychoeducation on this topic («And it may say in the books that with no sex outlet, you would have to be neurotic»), and sexual frustration («That you can say that the base of all is the sexual frustration and so forth»). |
| 43 Struggles with Self-Perception (20) | 'worse', 'dirty', 'you', 'it', 'felt', 'you as', 'awful', 'just', 'within yourself', 'felt that' | T addresses the negative aspects of C’s self-perception («Intellectually it might seem as though maybe you should find something very awful within yourself», «That everything inside of you is worse than you thought», «You really felt you had gotten a very dirty deal from nature»). |


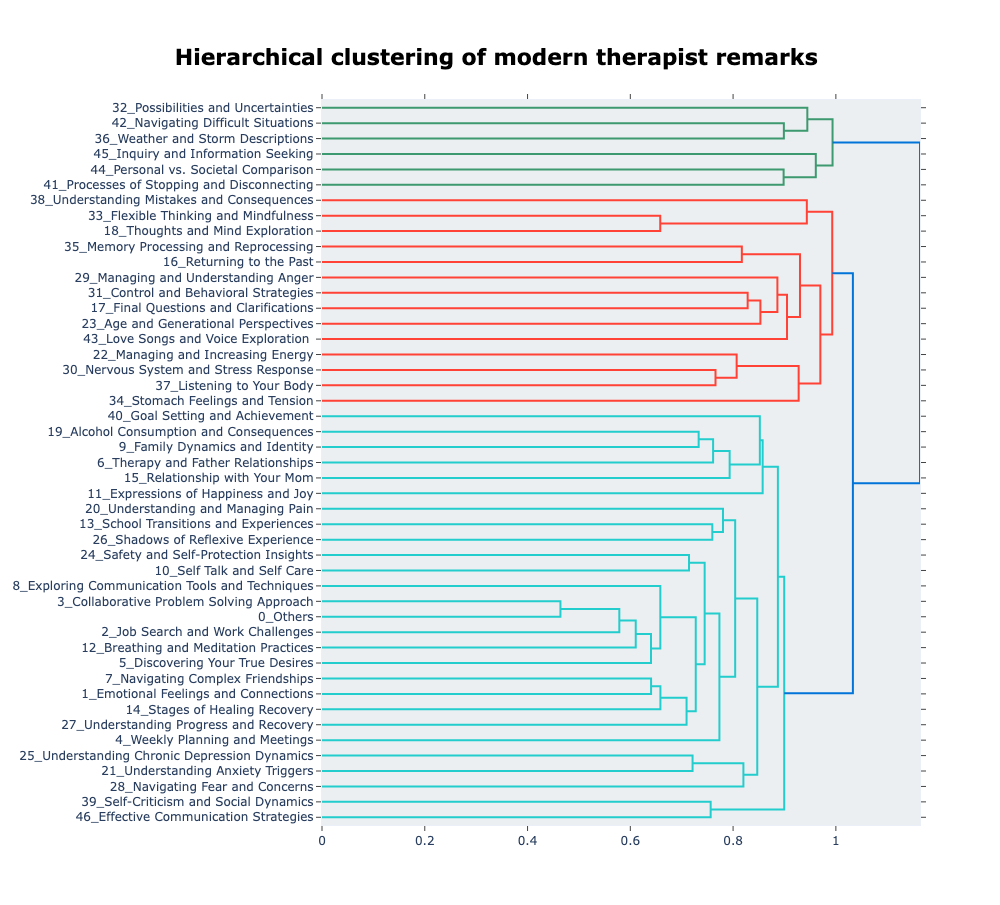


**Figure B2.** Results of hierarchical cluster analysis of modern therapist remarks

**Table B2.** Topics of Modern Therapists

| **Topic (Number of Objects)** | **Top 10 Keywords** | **Topic Description** |
| --- | --- | --- |
| 0 Others (4117) | 'she', 'not', 'okay', 'that', 'is', 'you', 'to', 'yeah', 'he', 'her' | This category contains various topics that were removed during the work process. |
| 1 Emotional Feelings and Connections (264) | 'feel', 'feeling', 'you feel', 'emotion', 'you', 'how', 'emotional', 'emotions', 'sadness', 'and' | T is interested in C’s current («Do you feel better?») or past state («How did you feel?»), his/her attitude toward something in a situation («How do you feel toward it as you noticed?»), as well as C’s attitude toward T («And how do you feel towards me?»). |
| 2 Job Search and Work Challenges (179) | 'job', 'work', 'you', 'income', 'that', 'working', 'to', 'your job', 'jobs', 'the job' | T discusses various aspects related to C’s job search («And let's go back to the job application and what you want to write down about that») or his/her current employment («Okay, I see you adjusted to the new income level with the full-time and part-time jobs together»). |
| 3 Collaborative Problem Solving Approach (174) | 'we', 'we can', 'we are', 'we have', 'to', 'we will', 'let', 'will', 'and', 'can' | T encourages C to engage in collaborative work and establishes the agenda for the therapy sessions («Do you know what we've been working on just to kind of make sure we're on the same page?», «So that's the kind of thing that the conflict you just described is the kind of thing that this works really well for and so that's what we could work on if you want»). |
| 4 Weekly Planning and Meetings (167) | 'week', 'time', 'minutes', 'next week', 'this week', 'next', 'morning', 'will', 'we', 'the' | T plans the session with C for next week («Same time next week») and also assigns some homework to C («So, consider those two things and we'll pick up with this next week»). |
| 5 Discovering Your True Desires (112) | 'do', 'want', 'you', 'you want', 'to do', 'need', 'do that', 'do you', 'that', 'what' | T addresses the topic of C’s self-determination («I mean there is a time when one wants to put one's energy into work and school and you know to figure out who we want to be in the world and what you want to do with your life»), including in the context of clarifying the goals of the therapy sessions («What is it that you need and we want to bring that into your life?»). |
| 6 Therapy and Father Relationships (108) | 'therapy', 'dad', 'therapist', 'your dad', 'counseling', 'father', 'your', 'your father', 'to', 'you' | T reflects on psychotherapy («I had one of my professors tell me he wants therapy to be unique in that it's two people focusing on one person») and addresses C’s relationship with his/her father («Were you close with your dad?»). |
| 7 Navigating Complex Friendships (106) | 'relationship', 'friend', 'friends', 'relationships', 'you', 'in', 'with', 'and', 'have', 'that' | T addresses the topic of friendship in C’s life («So, you said that you have a lot of friends who you've been talking to and meeting new people», «And then there's the relationship issue there»). |
| 8 Exploring Communication Tools and Techniques (102) | 'say', 'you say', 'you', 'tools', 'what', 'to say', 'system', 'experiment', 'say that', 'it' | T highlights C’s availability of various communication tools applicable to specific situations («And you got loads of different tools and techniques to help you do that»). |
| 9 Family Dynamics and Identity (102) | 'family', 'your family', 'the family', 'your', 'the', 'family and', 'and', 'as', 'you', 'with' | T discusses C’s relationships within his/her family («So just tell me a little bit about your family», «I would like you to frame the connection between you and your family»), including in the context of C’s identity («I mean we started from the topic of identity and where do I belong or where do I stay, so you made some comments on that and then we came to the family topic that, as far as I remember»). |
| 10 Self Talk and Self Care (90) | 'yourself', 'self', 'to yourself', 'self talk', 'you', 'to', 'would', 'myself', 'remind', 'about yourself' | T explores various aspects of C’s self-talk and self-care («What kind of things would you say to yourself?», «Would you feel comfortable giving that advice to yourself?»), while also expressing his/her acceptance of the client («I'm respecting and owning your current reality and accepting yourself in that rather than trying to persuade yourself that you have to be something other»). |
| 11 Expressions of Happiness and Joy (85) | 'happy', 'like', 'glad', 'joy', 'fun', 'am', 'like that', 'really', 'to hear', 'am so' | T encourages C by acknowledging his/her positive attitude («Yeah, you seem like a really happy person right now»), celebrates C’s progress in therapy («I'm really glad to hear that and it looks like you're sleeping better too»), and reflects on the topic of happiness and joy («You need to do things that bring you joy to be happy, but you need to be happy to be able to do that»). |
| 12 Breathing and Meditation Practices (85) | 'breathing', 'meditation', 'breath', 'you', 'yoga', 'to', 'practice', 'do', 'your', 'of' | T introduces breathing techniques and meditation («You notice more about your breathing when you are tuned to your breathing», «Meditation where you focus on just paying attention to your breath and what happens to all of that crazy thinking when you're just with your breath»), including in the context of helping C overcome anxiety («When you notice that anxiety is starting to heighten and then with your breathing you could do some of those other techniques that we talked about»). |
| 13 School Transitions and Experiences (83) | 'school', 'third', 'college', 'the school', 'class', 'the', 'teacher', 'school you', 'grade', 'in' | T discusses C’s educational experience, such as his/her forms of involvement in school activities («Did you do those activities in high school?») and C’s experiences attending different types of schools («How was that transition for you going from public school to private school?»). |
| 14 Stages of Healing Recovery (82) | 'recovery', 'healing', 'of', 'the', 'process', 'you', 'healing state', 'your', 'your recovery', 'state' | T notes that therapy is a gradual process and that there are different stages of recovery («And yeah, it sounds like to me you are at a different stage of recovery than where you were before», «And that's why I try to kind of push your way to recovery»). |
| 15 Relationship with Your Mom (81) | 'mom', 'your mom', 'your', 'mother', 'with your', 'your mother', 'her', 'mom and', 'with', 'you' | T explores C’s relationship with his/her mother («What effect would that have on your relationship with your mom?»), noting both the differences («That sounds like you and your mom were different») and similarities («Yeah, and you got that from your mom») between C and his/her mother. |
| 16 Returning to the Past (77) | 'back', 'go back', 'back to', 'go', 'history', 'past', 'back into', 'to the', 'back and', 'so' | T brings C back to a previously discussed issue («So let us go back to what we had before») or asks C what he/she would do if could return to a specific point in the past («If you could go back and…»). |
| 17 Final Questions and Clarifications (75) | 'questions', 'any', 'any questions', 'quiz', 'question', 'of questions', 'final questions', 'any final', 'any any', 'questions you' | T maintains the dialogue by asking C if he/she has any questions: before the start of the therapy session («All right, so I guess before we get started, do you have any questions for me?»), during it («You can talk about any questions»), and before its conclusion («Did any final questions that you got for now?»). |
| 18 Thoughts and Mind Exploration (74) | 'thought', 'think about', 'about', 'thinking', 'mind', 'think', 'thoughts', 'your mind', 'thinking about', 'what' | T asks C what he/she is thinking, both in general and in relation to the situation («What was going through your mind?», «What comes to you when you think about it?»), and also guides C to analyze the situation («Kind of thinking about that thought and what happens afterward»). |
| 19 Alcohol Consumption and Consequences (73) | 'drinking', 'alcohol', 'drink', 'you are', 'you', 'are', 'your', 'the', 'so', 'about' | T discusses C’s alcohol consumption («Okay, so to your wife, you're drinking is excessive, but to you, it is pretty normal»), the reasons behind it («The drinking has become a way for you to unwind and release some of the stress that you are feeling at work»), its impact on C («But when you're in the moment and drinking, it sounds like that's the only time that you said you’re feeling happy»), and the problems associated with C’s alcohol use («So, you're here because your wife feels that you have a problem with drinking»). |
| 20 Understanding and Managing Pain (70) | 'pain', 'painful', 'symptoms', 'the pain', 'the', 'physical symptoms', 'hurts', 'it', 'hurt', 'physical' | T discusses overcoming pain with C, primarily physical pain («You started feeling physical pain», «Seems like you're more or less making things worse in an effort to avoid the pain that you're going to suffer»). |
| 21 Understanding Anxiety Triggers (64) | 'anxiety', 'of anxiety', 'when', 'when you', 'that', 'of', 'the', 'panic', 'you', 'up' | T addresses anxiety, its causes, and C’s triggers that may provoke it («So, when you notice that anxiety comes up for you, where do you normally feel it?», «Just within the week when you notice that the anxiety came up because of that control reason»). |
| 22 Managing and Increasing Energy (60) | 'energy', 'the energy', 'the', 'of energy', 'of', 'and', 'your', 'more energy', 'more', 'you' | T discusses C’s energy («You're increasing your capacity that will bring more of your energy into your body») and notes that it needs to be managed effectively («Start investing a lot of energy», «That's what you need to do as the energy comes back fast; a real challenge is what to do with that energy at that point»). |
| 23 Age and Generational Perspectives (57) | 'old', 'how old', 'generations', 'old you', 'age', 'how', 'teenager', 'younger', 'you', 'young' | T asks about C’s current age («How old are you?») and also inquires about events in C’s life in relation to the age he/she was at the time those events occurred («How old were you when that started?», «So, you were how old when he passed away?»). |
| 24 Safety and Self-Protection Insights (49) | 'safe', 'safety', 'you', 'of', 'protectors', 'protect', 'to', 'the', 'that', 'protecting' | T discusses various aspects of C’s self-protection («What can you do that's going to give yourself a feeling of safety?», «And sometimes that ties in with, you know, there might be places that you can go to get safe»). |
| 25 Understanding Chronic Depression Dynamics (48) | 'depression', 'depressed', 'depression and', 'right', 'get', 'the', 'of depression', 'that', 'and', 'when' | T addresses the topic of depression and its causes («We're going to work on kind of getting to know about this depression and how it affects you and maybe the origins of it»), as well as various methods for dealing with it («As far as looking at the medications versus looking at the origins of the depression if maybe getting specific about when your last episode was to be able to see if we can get underneath, but we can take either path»). |
| 26 Shadows of Reflexive Experience (48) | 'it', 'it is', 'like', 'is', 'shadow', 'that', 'of', 'weird', 'kind of', 'kind' | T uses various forms of metaphor in his/her interpretations («So, it sounds like that part in the shadows is recognizing that this system», «It's something like a mist, like a mood that influences concrete things that come up in your mind», «It's kind of like sitting on a train watching the scenery go by and you're watching it»). |
| 27 Understanding Progress and Recovery (47) | 'progress', 'progress that', 'we', 'the', 'the progress', 'that', 'of', 'is', 'it is', 'what' | T discusses the importance of progress in therapy («The next thing from my point of view is when there's a little bit of progress», «But I think that's where I'm having real evidence of progress becomes important because if you're trying to kind of persuade yourself that you're better than you were»). |
| 28 Navigating Fear and Concerns (46) | 'fear', 'the fear', 'afraid', 'afraid of', 'worried', 'concern', 'about', 'the', 'worry', 'little concerned' | T discusses the topic of fear with C («What are you afraid of?») and its dangers to mental health («Can I tell you more about the danger of fear?»), as well as works with C’s fear using various techniques («Turn your attention to the Fear Part in your chest», «Yeah, let the fear know that you are not going to forget about it»). |
| 29 Managing and Understanding Anger (43) | 'angry', 'anger', 'your anger', 'anger and', 'consequence', 'you are', 'angry and', 'are', 'you', 'of' | T analyzes C’s anger response («What types of things do you think are triggering your anger response?»), the impact of anger on thinking and behavior («And it's hard when you're angry because when your anger is your focus on…»), and the possibilities for managing anger («In the consequence of that thought is I'm I'll be angry but I'm not going to act angry»). |
| 30 Nervous System and Stress Response (41) | 'nervous system', 'nervous', 'system', 'your nervous', 'stress', 'your', 'of', 'kind', 'stress response', 'kind of' | T discusses C’s nervous system («Do you know that that makes your nervous system?»), including its connection to emotions and stress («Do you feel like you're in a stressed-out overheating?»), and how to cope with them («My point is you want your nervous system to not be so agitated that you need something like to switch it off in the first place»). |
| 31 Control and Behavioral Strategies (32) | 'control', 'can', 'behavioral', 'behaviors', 'behavior', 'behavior so', 'not control', 'can not', 'we', 'think' | T discusses with C strategies and techniques for self-regulation of behavior («So that would be one behavioral strategy»), establishes boundaries of what can be controlled for C («We can only control what we're dealing with right here»), and engages in a conversation about the dichotomy of control («Do you think all three of those instances extend from the same thing of that something that I can't control like these things are outside of my control?»). |
| 32 Possibilities and Uncertainties (32) | 'possibility', 'maybe', 'probably', 'might', 'well', 'is possibility', 'well maybe', 'well it', 'it could', 'possible' | T considers various scenarios for the development of events in C’s situation («Right, that's one possibility», «Another possibility»). |
| 33 Flexible Thinking and Mindfulness (31) | 'thinking', 'mind', 'logical', 'of', 'thoughts', 'logical mind', 'wise mind', 'those thoughts', 'might', 'that' | T analyzes the thinking process with C («Before you didn't think to think about the thought that makes sense and there are a few steps nearby»), examining the influence of thoughts on feelings and behavior («But you could have those thoughts working for you»), as well as the process of evaluating cognitive acts («I'll learn how to evaluate my thinking, which might be a hundred percent true, or 0% true, or someplace in the middle»). |
| 34 Stomach Feelings and Tension (31) | 'stomach', 'your stomach', 'your', 'side of', 'in your', 'chest', 'side', 'tool', 'throat', 'your chest' | T works with C’s physiology, such as the abdominal muscles, to relieve tension («Let's see what it says your stomach muscles in your chest when you're angry», «Clench your stomach»). |
| 35 Memory Processing and Reprocessing (30) | 'memory', 'memories', 'target', 'bring up', 'the', 'up', 'the target', 'we', 'past', 'bring' | T refers to C’s memories («And I want you to think back to what is kind of like the earliest memory that you have of having that feeling or thought of I need to be in control») and also describes the process of memory work («And so, what that does is it really opens up the memory capsule or the memory storage and then that processing phase starts and what the reprocessing stage is essentially just to demystify; all it means is you're going to bring up all that material»). |
| 36 Weather and Storm Descriptions (30) | 'weather', 'wet', 'the weather', 'storm', 'water', 'rain', 'rihanna see', 'rihanna', 'today', 'mean' | T uses weather and storms as metaphors to describe C’s mood («How's the weather relating to you then?», «All I would want you to do is to remember the storm has a beginning, a middle, and an end»). |
| 37 Listening to Your Body (29) | 'body', 'your body', 'your', 'body and', 'and', 'the body', 'legs', 'in', 'listen to', 'arms and' | T notes the connection between the body and the mind («It's a way for your body trying to get your attention») and encourages C to listen to his/her body («I just really encourage you to listen to your body and work on your relationship with your body and soul»). |
| 38 Understanding Mistakes and Consequences (29) | 'mistake', 'mistakes', 'make mistake', 'make', 'consistent', 'in thinking', 'to make', 'thinking', 'everybody', 'they are' | T discusses the topic of mistakes, including cognitive errors («They're consistent errors in thinking»). T also normalizes C’s feelings («Everybody makes mistakes», «It’s reasonable that a professional makes a mistake like that»). |
| 39 Self-Criticism and Social Dynamics (29) | 'they', 'themselves', 'people', 'of', 'they are', 'or', 'that', 'kind of', 'kind', 'that they' | T explores the aspects of social dynamics in C’s life («But as people do, they have a way to kind of signal that they're not into that») and his/her dependence on the opinions of others («Do you have any evidence to do something that makes you think that they'll be critical of you?»). |
| 40 Goal Setting and Achievement (27) | 'goal', 'goals', 'your goal', 'oriented', 'goal for', 'your', 'your goals', 'you', 'reach', 'for' | T discusses C’s personal goals («You have a goal that you're trying to reach because your goal is to be a doctor, right?»), including those related to a specific therapy session («So, what would be your goal for today’s session?») and therapy as a whole («What would be your goal here?»). T also talks about the goal achievement process («I think that's important for us to note that planning is absolutely a part of what it would take for you to reach your goals»). |
| 41 Processes of Stopping and Disconnecting (27) | 'stop', 'shut', 'disconnect', 'stopped', 'stop the', 'down', 'shut down', 'because think', 'it down', 'because' | T explains the concept of stopping and disconnecting to C («This whole procedure is called stop»), manages the therapy session («Stop for a little bit», «Yeah, we probably should stop»), and suggests that C learn to pause his/her emotions in certain situations («Stop the feeling that automatically starts»). |
| 42 Navigating Difficult Situations (23) | 'difficult', 'hard', 'it is', 'easy', 'it', 'an easy', 'not an', 'very', 'is', 'boundaries' | T identifies the boundaries of the complexity of C’s situation («Yes, so it's a little more difficult than that»), normalizes C’s feelings regarding the situation's difficulty («It's a difficult thing to pull off»), and motivates C («Again, even if it is hard»). |
| 43 Love Songs and Voice Exploration (21) | 'song', 'voice', 'can', 'songs', 'sang', 'that song', 'hear you', 'hear', 'can you', 'this to' | T says that he/she hears C («I can hear you»), asks if C hears him/her («Tatiana, can you hear me?»), and also reflects on the topic of songs («Love songs that kind of inspiration YouTube thing at school and chemistry and just working stuff out»). |
| 44 Personal vs. Societal Comparison (21) | 'comparison', 'comparing', 'compare', 'personal', 'more of', 'the comparison', 'motivation', 'to compare', 'why', 'though you' | T discusses a topic such as C’s motivation in comparison to other people («And so, if you would like to stop comparing and we know the function of the comparison part of it and she is helping you it is effectively and that the motivation of you doing comparison», «Comparison because you're competitive you want to make sure that you are moving along with the rest and not getting lucky»). |
| 45 Inquiry and Information Seeking (20) | 'tell me', 'tell', 'me', 'about that', 'me about', 'about', 'so tell', 'to know', 'let', 'know about' | T maintains a dialogue with C and requests information from him/her («Yeah, so let's talk about that», «So, tell me a little bit about the…»). |
| 46 Effective Communication Strategies (20) | 'communication', 'they', 'the one', 'them', 'signals', 'how to', 'how', 'sending', 'to express', 'communicate' | T discusses with C issues related to building effective communication strategies («There is a pathway to communication», «Don't forget also to have you send out signals?»). |
