# Supplementary material for: Psychotherapist remarks’ ML classifier: insights from LLM and topic modeling application
Source: Front Psychiatry. 2025 Jul 25;16:1608163. doi: 10.3389/fpsyt.2025.1608163 (PMC12332746; doi:10.3389/fpsyt.2025.1608163)
Supplement: Supplementary file 3 [file SupplementaryFile3.docx]

Supplementary Material

Appendix C. Topic Structure of Merged Model

**Table C1.** Merged Topics of Classical and Modern Therapists

| **ID** | **Merged Topics** | **Topics of Classical Therapists** | **Topics of Modern Therapists** |
| --- | --- | --- | --- |
| 1 | Time Up and Future Meetings | 1 Time Management and Scheduling | 4 Weekly Planning and Meetings |
| 2 | Complex Emotions Toward Him | 2 Complex Emotions Toward Him | – |
| 3 | Desires and Disappointments | 3 Desires and Reluctance | 5 Discovering Your True Desires |
| 4 | Personal Growth and Decision-Making | 4 Personal Growth and Challenges | 33 Flexible Thinking and Mindfulness |
| 5 | Self-Acceptance and Relationships | 5 Self-Acceptance and Confidence Issues | 39 Self-Criticism and Social Dynamics |
| 6 | Understanding and Confronting Fear | 6 Exploring the Nature of Fear | 28 Navigating Fear and Concerns |
| 7 | See and Understanding Conversations | 7 Understanding the Concept of Seeing | – |
| 8 | Clarifying Meaning and Intent | 8 Clarifying Meaning in Conversations | 8 Exploring Communication Tools and Techniques |
| 9 | Desire to Escape and Leave | 9 Desire to Escape and Leave | – |
| 10 | Uncertainty and Understanding Issues | 10 Uncertainty and Clarity Issues | – |
| 11 | Open Conversation and Sharing | 11 Open Conversations About Feelings | 45 Inquiry and Information Seeking |
| 12 | Exploring Emotional Hurt and Bitterness | 12 Exploring Hurt and Bitterness | 20 Understanding and Managing Pain |
| 13 | Guilt and Self-Blame Dynamics | 13 Guilt and Self-Blame Dynamics | – |
| 14 | Dynamics of Meaningful Relationships | 15 Complexities of Relationships | 7 Navigating Complex Friendships |
| 15 | Struggles and Desires in Learning | 16 Personal Journey of Learning | 13 School Transitions and Experiences |
| 16 | Gender Roles and Relationships | 17 Gender Roles and Relationships | – |
| 17 | Struggles with Personal Change | 18 Struggles with Personal Change | 27 Understanding Progress and Recovery |
| 18 | Complex Mother-Sibling Relationships | 21 Mother-Daughter Relationship Dynamics | 15 Relationship with Your Mom |
| 19 | Voices and Perception of Sound | 23 Voice Perception and Despair | 43 Love Songs and Voice Exploration |
| 20 | Difficulties and Emotional Burdens | 24 Handling Difficult Dilemmas and Emotions | 42 Navigating Difficult Situations |
| 21 | Fear and Reflection on Aging | 26 Aging and Childhood Reflection | 23 Age and Generational Perspectives |
| 22 | Emotions of Crying and Tears | 28 Crying and Emotional Release | – |
| 23 | Father-Child Relationships and Authority | 29 Father-Child Relationships and Authority | 9 Family Dynamics and Identity |
| 24 | Possibilities and Potential Outcomes | 30 Possibilities and Potential Outcomes | 32 Possibilities and Uncertainties |
| 25 | Inner Struggle and Helplessness | 31 Inner Struggle and Helplessness | – |
| 26 | Pursuing Meaningful Personal Goals | 32 Understanding Personal Goals and Satisfaction | 40 Goal Setting and Achievement |
| 27 | Job Anxiety and Self-Reflection | 33 Fear and Anxiety About Work | 2 Job Search and Work Challenges |
| 28 | Marriage Anxiety and Dependence | 34 Marriage Doubts and Dependence | – |
| 29 | Expressions of Anger and Frustration | 37 Understanding and Expressing Anger | 29 Managing and Understanding Anger |
| 30 | Nurturing the inner child | 39 Embracing the Inner Child | – |
| 31 | Therapy and Father Relationships | – | 6 Therapy and Father Relationships |
| 32 | Expressions of Happiness and Joy | – | 11 Expressions of Happiness and Joy |
| 33 | Revisiting the Past Together | – | 16 Returning to the Past |
| 34 | Drinking Habits and Concerns | – | 19 Alcohol Consumption and Consequences |
| 35 | Managing and Increasing Energy Levels | – | 22 Managing and Increasing Energy |
| 36 | Understanding Depression and Its Roots | – | 25 Understanding Chronic Depression Dynamics |
| 37 | Nervous System and Stress Response | – | 30 Nervous System and Stress Response |

**Table C2.** Description of Topic Structure of Merged Model

| **Topic Group** | **Topic Name** | **Topic Description** |
| --- | --- | --- |
| I. Therapy Process and Communication (Topics related to the structure of therapy sessions, communication between therapist and client, and therapeutic goals) | 1. Time Up and Future Meetings | The topic includes both therapist’s (‘T’) statements about managing the session’s duration («*I see our time is up*») and remarks related to scheduling the next meeting («*Same time next week*»). T also assigns homework to the client (‘C’) («*So, consider those two things and we'll pick up with this next week*»). |
|  | 7. See and Understanding Conversations | T demonstrates his/her readiness to address the problematic situation («*Let's see*», «*But let's just look at that*») and shows understanding in response to C’s remarks («*I see*»), thereby supporting the dynamics of the therapeutic process. |
|  | 8. Clarifying Meaning and Intent | The topic contains T’s statements aimed at clarifying what C has said («*Is that what you mean?*», «*Is that what you're saying?*»). In this topic, T also highlights C’s availability of various communication tools applicable to specific situations («*And you got loads of different tools and techniques to help you do that*»). |
|  | 11. Open Conversation and Sharing | The topic contains T’s statements intended to begin the therapy session with C («*Now, what I would like would be for you to tell me anything you’re willing to tell me about yourself and your situation*»), as well as other requests for information from T to C («*Tell me, what's your first name?*», «*Can you tell me any more of your thinking about it?*», «*Yeah, so let's talk about that*», «*So, tell me a little bit about the…*»). |
|  | 19. Voices and Perception of Sound | T metaphorically addresses C’s inner voice («*I guess another puzzling thing about it is that you feel that you hadn’t heard the voice for quite a while, why, why would it return?*», «*And was it the voice that said, ‘If you are feeling desperate...’*»), also noting the despair in C’s tone («*Oh, the voice sounded kind of desperate*»). T also says that he/she hears C («*I can hear you*»), asks if C hears him/her («*Tatiana, can you hear me?*»). This topic also includes T’s statements about the volume of C’s voice («*I didn't quite hear what you said*»), and reflects on the topic of songs («*Love songs that kind of inspiration YouTube thing at school and chemistry and just working stuff out*»). |
|  | 31. Therapy and Father Relationships | T reflects on psychotherapy («*I had one of my professors tell me he wants therapy to be unique in that it's two people focusing on one person*») and also explores C’s relationship with his/her father («*Were you close with your dad?*»). |
| II. Emotions, Fears, and Emotional Regulation (Topics exploring the client's emotions, including fear, anger, sadness, joy, and stress, as well as coping mechanisms) | 2. Complex Emotions Toward Him | T examines C’s relationship with an abstract male figure («*You feel he has a dark critic inside of him*», «*There are certain things you respect him for, but that doesn’t, uh, alter the fact that you definitely hate him and don't love him*»). |
|  | 6. Understanding and Confronting Fear | T discusses the topic of fear with C («*What are you afraid of?*») and its dangers to mental health («*Can I tell you more about the danger of fear?*»). T also addresses the nature of C’s fear, including its various forms («*They nevertheless are fears deep inside, and the biggest fear of all is the fear of being trapped, in so many different ways*», «*So that's where you fear is, a fear of a relationship with a man*», «*It really wasn’t the fear of death; that you can accept*», «*Fear of choking, fear of convulsions?*», «*It seems to me that you're saying, ahh, the fear, the fears grow stronger, as time goes by, both of marriage and of children and of commitment, as well as the fear of aging... that it seems a package of fears*»).T attempts to normalize C’s fear («*The fear comes and goes*», «*To know that you are not alone in that fear, the others have the same kind of fear*») and interprets C’s anxious state («*The more clear our feeling of fear that exists in us here is blocked off close to you, that then somehow it develops into this more nameless, more indescribable kind of fear that is anxiety*»). T also works with C’s fear using various techniques («*Turn your attention to the Fear Part in your chest*», «*Yeah, let the fear know that you are not going to forget about it*»). |
|  | 20. Difficulties and Emotional Burdens | T addresses the dilemmas and difficulties in C’s life, possibly with the intention of normalizing C’s emotions regarding the complexity of the situation («*It is a real dilemma*», «*For you, you feel it's almost too much, is that it?*», «*Yes, so it's a little more difficult than that*», «*It's a difficult thing to pull off*»). T also motivates C («*Again, even if it is hard*»). |
|  | 21. Fear and Reflection on Aging | T asks about C’s current age («*How old are you?*») and inquires about events in C’s life in relation to the age C was at the time those events occurred («*How old were you when that started?», «So, you were how old when he passed away?*»). T also explores C’s world through the lens of childhood and aging («*You may behave in a certain way, and then only later realize that that was a step from childhood into adolescence*») and addresses C’s fear of growing old («*Can you tell me a little bit more about your fear that you have of aging?*»). |
|  | 22. Emotions of Crying and Tears | T discusses with C sensitive topics that may evoke tears («*It almost brings tears to your eyes, doesn't it?*»), also noting C’s tears («*There are tears now*») and normalizing C’s feelings about them, thereby expressing empathy («*Yes, you could cry*»). |
|  | 29. Expressions of Anger and Frustration | T explores the nature of C’s anger. Specifically, T notes anger in C’s speech («*I can hear the anger in there*»), analyzes C’s anger response («*What types of things do you think are triggering your anger response?*»), and examines the impact of anger on thinking and behavior («*And it's hard when you're angry because when your anger is your focus on…*»). T legitimizes C’s desire to feel anger («*If you feel like being angry, you can be angry*»), and discusses the possibilities for managing anger («*In the consequence of that thought is I'm I'll be angry but I'm not going to act angry*», («*So, when you meet anger, whether in men or women, you tend to placate it if possible*»). |
|  | 32. Expressions of Happiness and Joy | T encourages C by acknowledging his/her positive attitude («*Yeah, you seem like a really happy person right now*»), celebrates C’s progress in therapy («*I'm really glad to hear that and it looks like you're sleeping better too*»), and reflects on the connection between happiness and joy («*You need to do things that bring you joy to be happy, but you need to be happy to be able to do that*»). |
|  | 37. Nervous System and Stress Response | T discusses C’s nervous system («*Do you know that that makes your nervous system?*»), including its connection to emotions and stress («*Do you feel like you're in a stressed-out overheating?*»), and how to cope with them («*My point is you want your nervous system to not be so agitated that you need something like to switch it off in the first place*»). |
| III. Self-Perception, Identity, and Inner Conflict (Topics related to self-esteem, self-acceptance, identity, internal conflicts, and guilt) | 5. Self-Acceptance and Relationships | T explores C’s confidence and lack of confidence. For instance, T notes C’s confidence in his/her feelings («*You feel kind of a confidence in your own feelings*»), discusses potential reasons for C’s sense of inadequacy («*You feel that something should have turned up to give you that confidence in yourself*»), and analyzes aspects of C’s self-perception in relation to how he/she is perceived by others («*You feel that you are living by the standards others have and what they think of you and so on, even though more deeply you know that you can't possibly have happiness that way*»). T also explores aspects of social dynamics in C’s life («*But as people do, they have a way to kind of signal that they're not into that*») and C’s dependence on the opinions of others («*Do you have any evidence to do something that makes you think that they'll be critical of you?*»). |
|  | 13. Guilt and Self-Blame Dynamics | T addresses C’s sense of guilt («*You feel guilty about what you haven't done, and it gives you more reason for avoiding the people*»), tries to explain the origins of this sense («*You don’t like to blame your school or your family, but still you feel that to some extent your family was responsible*»), and explores C’s self-blame («*That sounds like not only your family looks down on it or something, but that you scold yourself for it too*»). |
|  | 16. Gender Roles and Relationships | T examines C’s gender roles. Specifically, T interprets C’s issues («*So, it's really a big problem for you, you are feminine and you like to be feminine and you're seen as feminine and reacted to in that way, and then you think, ‘Oh my God!’*», «*When you feel insecure about the better sexual relationship, it might be because it hits you kind of hard that that stresses the fact that you are a woman with a woman's needs*») and notes C’s masculinity («*You see both elements of yourself pretty sharply, where on the one hand you may be more of a woman than you think you are, and on the other hand you have pretty masculine interests along some lines*»). |
|  | 17. Struggles with Personal Change | T addresses C’s personal changes, touching on aspects such as C’s procrastination («*You think to make a change you ought to change right now; on the other hand, you think that maybe it would have been simpler to delay and change next quarter*») and interpreting the difficulties with change that C experiences («*But you're just very much aware of a loosening type of change taking place, and intellectually when you can stand off and look at it to see, if...you knew exactly what made the wheels go round, hm?*», «*You tend to dwell a little more on what you didn't do in the past, rather than on what might be done right now or in the future*»). T also discusses the importance of progress in therapy («*The next thing from my point of view is when there's a little bit of progress*», «*But I think that's where I'm having real evidence of progress becomes important because if you're trying to kind of persuade yourself that you're better than you were*»). |
|  | 25. Inner Struggle and Helplessness | T explores C’s inner struggle («*You're inclined to feel that war situation or no war situation, the struggle is pretty much within you, after all*»), C’s sense of helplessness («*You feel that it's really the back of the coming in is the fact that you feel helpless to do anything about it*»), potential strategies for overcoming this feeling («*In other words, when you begin to feel hopeless then it seems so necessary to distract yourself from yourself*»), and the possibility of external support («*It's pretty deeply annoying to get into the conflicts and then not be sure which way to go and wish like hell somebody would give a little push*»). |
|  | 30. Nurturing the Inner Child | T metaphorically and with a touch of irony addresses C, urging him/her to pay attention to his/her inner self («*You know that little girl is inside of you*», «*The naughty little girl can get away with things*»), while also reflecting on the process of growing up («*The little girl, the little girl will grow up if you care enough for her*»). |
| IV. Relationships and Social Dynamics (Topics exploring the client's relationships with family, friends, partners, and colleagues, as well as social interactions) | 14. Dynamics of Meaningful Relationships | T analyzes various aspects of C’s relationships with others («*You know that it’s a difficult relationship, and you’d like to preserve it, but it’s going to take a great deal of energy on your part to hold that relationship together*»), including possible strategies for improvement («*It doesn't spoil the relationship to say ‘No’*»). T also addresses the topic of friendship in C’s life («*So, you said that you have a lot of friends who you've been talking to and meeting new people*», «*And then there's the relationship issue there*»). |
|  | 18. Complex Mother-Sibling Relationships | T analyzes C’s relationship with mother («*It makes you feel as though…you are a mouse in your relationship to your mother*», «*What effect would that have on your relationship with your mom?*»), noting both the differences («*That sounds like you and your mom were different*») and similarities («*Yeah, and you got that from your mom*», «*So, you’re saying your mother was the model for this organizing self*») between C and mother. |
|  | 23. Father-Child Relationships and Authority | T analyzes various facets of C’s relationship with father or stepfather («*That things went somewhat better between you and your father*», «*Just like your father*»). T also discusses C’s relationships within family («*So just tell me a little bit about your family*», «*I would like you to frame the connection between you and your family*»), including in the context of C’s identity («*I mean we started from the topic of identity and where do I belong or where do I stay, so you made some comments on that and then we came to the family topic that, as far as I remember*»). |
|  | 28. Marriage Anxiety and Dependence | T examines C’s relationship with his/her life partner, addressing aspects such as C’s feelings in the marriage («*You’re not happy with your husband and he’s not happy with you?*») and the fear of commitment («*There is a fear of commitment, and a fear of having children. And a feeling that in marriage you don't want to give up your identity*»). T also explores C’s hidden reasons for entering into marriage («*Perhaps one of the things you looked forward to in marriage was that there would be a situation and a person on which you could basically depend*»). |
| V. Personal Growth, Life Direction, and Decision-Making (Topics related to personal development, finding life direction, decision-making, and exploring possibilities) | 3. Desires and Disappointments | T identifies desirable and undesirable scenarios for C («*You don’t want to make it any darker than it is*», «*You don’t want to starve and you don’t want to cross the Alps*»). T also addresses C’s self-determination («*I mean there is a time when one wants to put one's energy into work and school and you know to figure out who we want to be in the world and what you want to do with your life*»), including in the context of clarifying the goals of therapy («*What is it that you need and we want to bring that into your life?*»). |
|  | 4. Personal Growth and Decision-Making | T addresses C’s lack of clarity regarding life direction («*You don't know at all where you want to, what direction you want to move or what you want to do*»). T also interprets C’s indecision in choosing a life path («*You feel that on the one hand you're not living up to all the things you really should be doing, but on the other hand, you feel that it's more realistic to grow gradually into that*») and highlights C’s progress in personal growth («*I feel that you’ve made a good deal of progress inside yourself*»). T also analyzes the thinking process with C («*Before you didn't think to think about the thought that makes sense and there are a few steps nearby*»), examining the influence of thoughts on feelings and behavior («*But you could have those thoughts working for you*»), as well as the process of evaluating cognitive acts («*I'll learn how to evaluate my thinking, which might be a hundred percent true, or 0% true, or someplace in the middle*»). |
|  | 9. Desire to Escape and Leave | T explores both C’s desire to escape the situation he/she finds himself/herself in («*Seems as though the thing that is stirring within you is more 'I want to get away, be out on my own, be free'*») and the difficulties that accompany this («*And you know what a struggle it was to move away from that*», «*Just felt that there was a need to get away and you couldn't explain it to others; you just couldn't*»). |
|  | 10. Uncertainty and Understanding Issues | T acknowledges a lack of understanding of C’s situation («I'm not quite sure»), and also notes the uncertainty in C’s words («You're not quite sure why»). |
|  | 24. Possibilities and Potential Outcomes | T draws C’s attention to various possible outcomes and scenarios for the development of events in C’s situation («*It’s one possible option at any rate*», «*Yes, probably that would be another possible outcome*», «*Right, that's one possibility*», «*Another possibility*»). |
|  | 26. Pursuing Meaningful Personal Goals | T examines C’s personal goals («*You have a goal that you're trying to reach because your goal is to be a doctor, right?*»), delving into the nature of these goals («*In other words, the goal as nearly as you can formulate it is some kind of fusion between the things intellectually you know you want and something pretty deep in you that doesn't lend itself easily to words, or labels*...»). T highlights the connection between goal achievement and self-knowledge («*Whereas the kind of goal you want to reach probably can be more easily achieved when you understand some aspects of yourself*»). T assesses C’s progress in reaching personal goals («*It’s just the gradual realization that you are not as far toward the goal as you hoped*») and identifies the necessary conditions for achieving these goals («*You expected to reach the goal without the work or struggle that went in between*»). T emphasizes the importance of planning in the goal achievement process (*«I think that's important for us to note that planning is absolutely a part of what it would take for you to reach your goals»*). T also sets goals for individual therapy sessions (*«So, what would be your goal for today’s session?»*). |
| VI. Work, Education, and Career (Topics related to the client's professional life, education, and career aspirations) | 15. Struggles and Desires in Learning | T explores C’s desires in the areas of learning and education («*You feel a real desire to try to learn some of this for what you would like to learn from it, not in terms of meeting some future examination demand or any other future demand*», «*You feel that you really like to achieve and learn a lot, but you just, you're just not doing it at present*»). T also discusses C’s educational experience, such as C’s forms of involvement in school activities («*Did you do those activities in high school?*») and C’s experiences attending different types of schools («*How was that transition for you going from public school to private school?*»). |
|  | 27. Job Anxiety and Self-Reflection | T discusses various aspects related to C’s job search («*And let's go back to the job application and what you want to write down about that*») or C’s current employment («*Okay, I see you adjusted to the new income level with the full-time and part-time jobs together*»). T also addresses C’s negative emotions related to work («*You feel as though staying in the job situation may really bring a blow-up on your part*»), and explores C’s self-reflection regarding work («*That is, sort of asking yourself, why the hell should I be fearful of or overwhelmed by the notion of a job?*»). |
| VII. Past Experiences and Their Influence (Topics analyzing past experiences and their impact on the client's current state) | 12. Exploring Emotional Hurt and Bitterness | T explores the area of C’s emotional pain and bitterness. Specifically, T identifies the presence of painful feelings («*It looks like you’re feeling some of that hurt right now*»), analyzes their causes («*That’s the way you have been hurt*»), emphasizes the importance of reflection on pain («*It's been so good to get out both the hurt and the bitterness where you can look at them*»), and explains the different facets of pain to C («*There’s a lot of pain there – pain about the past and pain about the present*»). T also discusses with C overcoming pain («*Seems like you're more or less making things worse in an effort to avoid the pain that you're going to suffer*»). |
|  | 33. Revisiting the Past Together | T brings C back to a previously discussed issue («*So let us go back to what we had before*») or asks C what he/she would do if could return to a specific point in the past («*If you could go back and…*»). |
| VIII. Health and Well-Being (Topics related to physical and mental health, including depression, alcohol use, and energy management) | 34. Drinking Habits and Concerns | T discusses C’s alcohol consumption («*Okay, so to your wife, you're drinking is excessive, but to you, it is pretty normal*»), the reasons behind it («*The drinking has become a way for you to unwind and release some of the stress that you are feeling at work*»), its impact on C («*But when you're in the moment and drinking, it sounds like that's the only time that you said you’re feeling happy*»), and the problems associated with C’s alcohol use («*So, you're here because your wife feels that you have a problem with drinking*»). |
|  | 35. Managing and Increasing Energy Levels | T discusses C’s energy («*You're increasing your capacity that will bring more of your energy into your body*») and notes that it needs to be managed effectively («*Start investing a lot of energy*», «*That's what you need to do as the energy comes back fast; a real challenge is what to do with that energy at that point*»). |
|  | 36. Understanding Depression and Its Roots | T addresses the topic of depression and its causes («*We're going to work on kind of getting to know about this depression and how it affects you and maybe the origins of it*»), as well as exploring various methods for dealing with it («*As far as looking at the medications versus looking at the origins of the depression if maybe getting specific about when your last episode was to be able to see if we can get underneath, but we can take either path*»). |
