# Supplementary material for: Psychotherapist remarks’ ML classifier: insights from LLM and topic modeling application
Source: Front Psychiatry. 2025 Jul 25;16:1608163. doi: 10.3389/fpsyt.2025.1608163 (PMC12332746; doi:10.3389/fpsyt.2025.1608163)
Supplement: Supplementary file 4 [file SupplementaryFile4.docx]

Supplementary Material

Appendix D. Ranked distribution of topics

**Table D1.** Ranked distribution of topics for Carl Rogers' sessions and cognitive-behavioral therapy (CBT) sessions of modern therapists

| Topic | Topic name | Rank (C. Rogers) | Rank (CBT) |
| --- | --- | --- | --- |
| 1 | Time Up and Future Meetings | 2 | 1 |
| 2 | Complex Emotions Toward Him | 14 | 13 |
| 3 | Desires and Disappointments | 1 | 3 |
| 4 | Personal Growth and Decision-Making | 5 | 17.5 |
| 5 | Self-Acceptance and Relationships | 12.5 | 17.5 |
| 6 | Understanding and Confronting Fear | 10 | 15 |
| 7 | See and Understanding Conversations | 3 | 2 |
| 8 | Clarifying Meaning and Intent | 7 | 7 |
| 9 | Desire to Escape and Leave | 7 | 13 |
| 10 | Uncertainty and Understanding Issues | 15 | 17.5 |
| 11 | Open Conversation and Sharing | 9 | 13 |
| 12 | Exploring Emotional Hurt and Bitterness | 21 | 23.5 |
| 13 | Guilt and Self-Blame Dynamics | 19 | 31 |
| 14 | Dynamics of Meaningful Relationships | 31.5 | 7 |
| 15 | Struggles and Desires in Learning | 29.5 | 27.5 |
| 16 | Gender Roles and Relationships | 28 | 35.5 |
| 17 | Struggles with Personal Change | 19 | 23.5 |
| 18 | Complex Mother-Sibling Relationships | 33 | 17.5 |
| 19 | Voices and Perception of Sound | 25 | 31 |
| 20 | Difficulties and Emotional Burdens | 7 | 9 |
| 21 | Fear and Reflection on Aging | 25 | 31 |
| 22 | Emotions of Crying and Tears | 17 | 35.5 |
| 23 | Father-Child Relationships and Authority | 27 | 23.5 |
| 24 | Possibilities and Potential Outcomes | 4 | 4.5 |
| 25 | Inner Struggle and Helplessness | 16 | 7 |
| 26 | Pursuing Meaningful Personal Goals | 29.5 | 35.5 |
| 27 | Job Anxiety and Self-Reflection | 25 | 4.5 |
| 28 | Marriage Anxiety and Dependence | 36 | 31 |
| 29 | Expressions of Anger and Frustration | 22 | 23.5 |
| 30 | Nurturing the inner child | 19 | 23.5 |
| 31 | Therapy and Father Relationships | 23 | 35.5 |
| 32 | Expressions of Happiness and Joy | 12.5 | 10 |
| 33 | Revisiting the Past Together | 11 | 11 |
| 34 | Drinking Habits and Concerns | 35 | 31 |
| 35 | Managing and Increasing Energy Levels | 31.5 | 27.5 |
| 36 | Understanding Depression and Its Roots | 37 | 20 |
| 37 | Nervous System and Stress Response | 34 | 23.5 |
